# The CSN/COP9 Signalosome Regulates Synaptonemal Complex Assembly during Meiotic Prophase I of *Caenorhabditis elegans*

**DOI:** 10.1371/journal.pgen.1004757

**Published:** 2014-11-06

**Authors:** Heather Brockway, Nathan Balukoff, Martha Dean, Benjamin Alleva, Sarit Smolikove

**Affiliations:** 1 Interdisciplinary Program in Genetics, University of Iowa, Iowa City, Iowa, United States of America; 2 Department of Biology, University of Iowa, Iowa City, Iowa, United States of America; University of California, Davis, United States of America

## Abstract

The synaptonemal complex (SC) is a conserved protein structure that holds homologous chromosome pairs together throughout much of meiotic prophase I. It is essential for the formation of crossovers, which are required for the proper segregation of chromosomes into gametes. The assembly of the SC is likely to be regulated by post-translational modifications. The CSN/COP9 signalosome has been shown to act in many pathways, mainly via the ubiquitin degradation/proteasome pathway. Here we examine the role of the CSN/COP9 signalosome in SC assembly in the model organism *C. elegans*. Our work shows that mutants in three subunits of the CSN/COP9 signalosome fail to properly assemble the SC. In these mutants, SC proteins aggregate, leading to a decrease in proper pairing between homologous chromosomes. The reduction in homolog pairing also results in an accumulation of recombination intermediates and defects in repair of meiotic DSBs to form the designated crossovers. The effect of the CSN/COP9 signalosome mutants on synapsis and crossover formation is due to increased neddylation, as reducing neddylation in these mutants can partially suppress their phenotypes. We also find a marked increase in apoptosis in *csn* mutants that specifically eliminates nuclei with aggregated SC proteins. *csn* mutants exhibit defects in germline proliferation, and an almost complete pachytene arrest due to an inability to activate the MAPK pathway. The work described here supports a previously unknown role for the CSN/COP9 signalosome in chromosome behavior during meiotic prophase I.

## Author Summary

Meiosis is a cellular division required for the formation of gametes, and therefore sexual reproduction. Accurate chromosome segregation is dependent on the formation of crossovers, the exchange of DNA between homologous chromosomes. A key process in the formation of crossovers is the assembly of the synaptonemal complex (SC) between homologs during prophase I. How functional SC structure forms is still not well understood. Here we identify CSN/COP9 signalosome complex as having a clear role in chromosome synapsis. In CSN/COP9 mutants, SC proteins aggregate and fail to properly assemble on homologous chromosomes. This leads to defects in homolog pairing, repair of meiotic DNA damage and crossover formation. The data in this paper suggest that the role of the CSN/COP9 signalosome is to prevent the aggregation of central region proteins during SC assembly.

## Introduction

The formation of haploid gametes is critical for reproduction in most eukaryotic organisms. Meiosis is the specialized cellular division leading to the formation of gametes, which in metazoans are eggs and sperm. Unlike mitosis, meiosis has one round of chromosome replication followed by two divisions: the first division is referred to as MI, in which homologous chromosomes segregate from each other, and the second division is referred to as MII, where sister chromatids segregate. It is essential that chromosome segregation during the divisions occurs correctly or an aberrant number of chromosomes will be present in the gametes, resulting in aneuploid eggs or sperm and consequently aneuploid or inviable offspring [1].

In meiotic prophase I, preceding the first division, homologous chromosomes pair, synapse, and form crossovers to recombine the genetic material. Crossovers and sister chromatid cohesion result in chiasmata, the visually detectable connections between homologous chromosomes observed in late prophase I. Chiasmata allow homologs to align properly at the metaphase plate during meiosis I and subsequently segregate to opposite poles [2]. All prophase I steps are highly regulated, ensuring that meiotic prophase proceeds correctly.

The synaptonemal complex (SC) is an evolutionarily conserved protein structure connecting pairs of homologous chromosomes during most prophase I stages and is required for formation of most crossovers [3]. Absent or improperly formed SC inhibits crossover formation, resulting in missegregation of chromosomes [4]. The SC is composed of lateral element proteins, which bind to the chromosomal axis of each homolog. The lateral element proteins are connected by the central region (CR) proteins, forming a physical link which holds homologous chromosomes together throughout most of meiotic prophase I [5]–[8]. In *C. elegans*, lateral element proteins include HTP-1/2, HTP-3, and HIM-3 [5]–[10]; there are four known CR proteins: SYP-1, SYP-2, SYP-3, and SYP-4 (collectively known as SYPs). The SYPs act in an interdependent manner: if one is missing, the CR does not form. The phenotypic consequences of mutations in all four SYPs are indistinguishable: lack of synapsis and failure to form crossovers [11]–[13].

In certain mutants, CR proteins can also assemble into aberrant SC-like structures that are non-functional and do not support crossover formation. In *C. elegans*, CR components are found to assemble between non-homologous chromosomes (non-homologous synapsis [7], [9] or sisters [11], [14]). CR proteins can also self-aggregate, forming polycomplexes (PCs). By electron microscopy, PCs are reminiscent of SC structures and in most cases, they are not associated with DNA [15]. Although PCs can contain multiple SC proteins, single components of the CR can form PCs without the aid of lateral element proteins [16]. PCs can be found in wild-type meiotic cells, when the SC assembles or disassembles, but these are small structures that are tightly regulated [17]. In some aberrant situations, large and persistent PCs are observed, indicating that in the absence of proper regulation CR proteins have a natural propensity to aggregate. These structures are found in tissue culture cells where CRs are expressed ectopically [18] and frequently found in yeast meiotic mutants [19]. In *C. elegans*, there are four examples for large and persistent PC-like structures (upon SC assembly [14], [20], [21] or disassembly [22]). The molecular mechanism leading to PC formation in these mutants is unknown.

Pathways regulating SC assembly to prevent PCs may be different between yeast meiosis and meiosis in other organisms. When recombination or SC assembly is perturbed, the yeast CR protein Zip1 readily forms PCs. On the contrary, none of the *C. elegans* CR proteins/SYPs aggregate when some SC proteins are missing or recombination fails [11]–[13], [23] These observations raise the possibility that CR proteins self-aggregation (*i.e.*, form PCs) is more tightly regulated in *C. elegans* meiosis.

In yeast and mouse, lateral element proteins have been shown to be post-translationally modified via sumoylation and phosphorylation which affects SC morphogenesis [24], [25]. Proper SC assembly may also involve post-translational modifications of CR proteins. In *C. elegans*, it is not known if such mechanisms exist and how CR proteins are post-translationally modified.

The CSN/COP9 signalosome is a highly conserved protein complex involved in post-translational modifications, originally described in *Aradidopsis* as a repressor of photomorphogenesis [26]. The complex is comprised of eight subunits which are similar to the lid complex of the 26S proteasome [27], [28]. Seven CSN/COP9 signalosome subunits have been identified in *C. elegans*. Five subunits (CSN-1,2,3,4, and 7) contain a PCI (**p**roteasome, **C**OP9 signalosome, **i**nitiation factor 3) domain and two (CSN-5 and CSN-6) contain MPN (**M**pr1-**P**ad1-**N**-terminal) domains [28]. The PCI domains are thought to facilitate protein-protein interactions and may also have nucleic acid binding properties [29]. The CSN-5 MPN domain contains a JAMM (**Ja**b1/**M**PN/**M**ov34) motif, which includes the metalloisopeptidase catalytic activity, which can cleave ubiquitin and ubiquitin-like post-translational modifiers (such as NED-8/Rub1) [30]–[33]. The CSN-6 MPN domain lacks the JAMM motif and thus the metalloisopeptidase activity [34], [35]. The signalosome is involved in the regulation of protein function via multiple pathways, but most studies have been carried out in the context of ubiquitin pathway via the CULLIN-RING E3 ubiquitin ligases (CRLs) [27], [32], [36]. The signalosome, through deneddylation of the CRLs, down-regulates proteasome degradation and/or monoubiquitination of substrates [36]–[38]. This deneddylation activity occurs in the context of the signalosome holocomplex. The CSN/COP9 signalosome affects cell cycle, gene expression, and DNA damage repair, through mechanisms that do not necessarily involve CRLs [39]–[41]. The understanding of the role of the CSN/COP9 signalosome in meiosis is limited. In *Drosophila*, the CSN/COP9 signalosome is required for meiotic progression [42]. A recent study in *Arabidopsis* identified a role for neddylation in crossover distribution and SC assembly, but the CSN/COP9 signalosome was not yet examined in this context [43].

Null mutants of the CSN/COP9 signalosome generated in other model organisms (yeast and *Drosophila*) have shown that the loss of one subunit renders it inactive and leads to lethality [44]–[47]. CSN-5 (also known as Jab1) has been shown to act outside the holocomplex in such cellular activities as nuclear export, degradation, and protein stabilization [48], [49]. The CSN-5 subunit of CSN/COP9 signalosome in *C. elegans* has also been implicated in muscle development [50], and the regulation of germline P-granule component, GLH-1, through interactions with KGB-1, a member of the JNK kinase family [51], [52]. While CSN-5 RNAi has been shown to reduce the size of gonads in *C. elegans* [51], [52], a role for CSN-5 in meiotic chromosome behavior has not been examined.

The work described here indicates a novel role for the CSN/COP9 signalosome in meiotic prophase I events that are key for the formation of functional gametes. Mutations in signalosome components specifically affected SC assembly and oocyte maturation. In *csn* mutants SYP-1 aggregated (PC-like structures) formed and persisted throughout meiotic prophase I. Additionally, we observed reduced chromosomal pairing throughout meiotic prophase as well as disruption in meiotic recombination and crossover formation. The defects in crossover formation were partially suppressed by reducing the levels of neddylation or ubiquitination. We also found an increase in apoptosis, likely due to the disruption of events earlier in pachytene. Oocyte maturation also was disrupted, leading to a severe reduction in the number of oocytes in diakinesis, which rendered the worms sterile. Our working model is that the CSN/COP9 complex regulates SC morphogenesis by inhibiting SYP DNA-independent self-assembly. Without CSN/COP9 function SC morphogenesis is perturbed (leading to CR aggregate formation) as are subsequent downstream events (*e.g.*, pairing and recombination) which are dependent on proper SC formation. Furthermore, the CSN signalosome affects oocyte maturation and permitting meiotic progression via MAPK/MPK-1 activation.

## Results

### *csn* mutants exhibit defects in SC morphogenesis and meiotic progression

We identified *csn-5* as a gene involved in SC morphogenesis via an RNAi suppressor/enhancer screen of a mutant (*akir-1*) exhibiting aberrant SC aggregation. Previous studies utilizing RNAi methodology to examine the role of the CSN complex genes in *C. elegans* demonstrated that *csn-5* was required for normal gonad morphology. *csn-5(RNAi)* resulted in formation of short gonads and down-regulation of the P-granule component GLH-1 [51], [52]. However, SC morphogenesis, chromosome dynamics, or meiotic recombination in meiotic prophase I were not examined in these studies. Here we focused our studies on the function of the CSN/COP9 signalosome in these meiotic processes.

**Figure 1:**
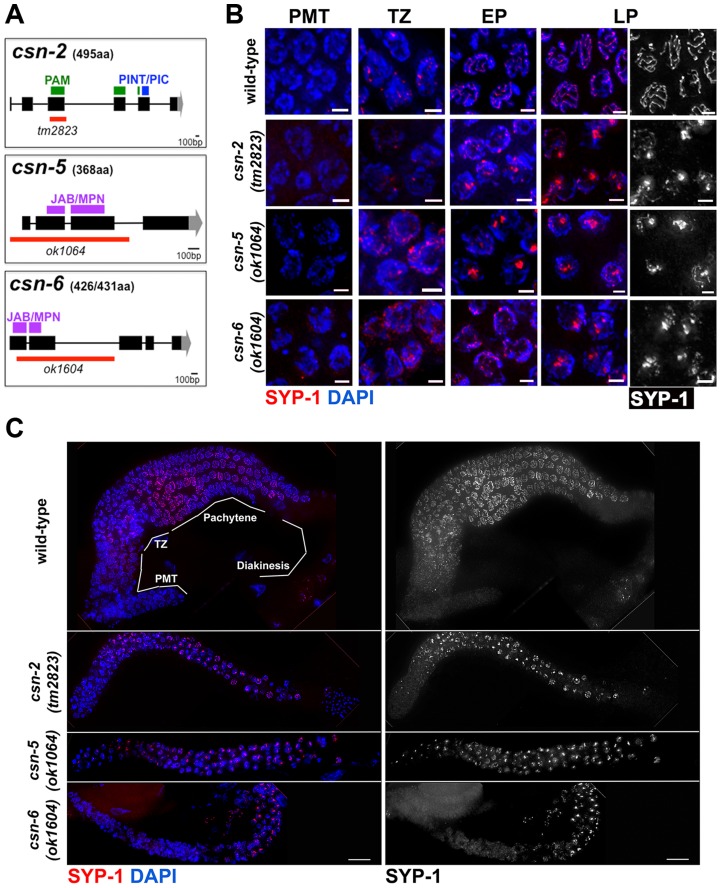
SC central element assembly defects in *csn* mutants. A) CSN alleles used in this study: black rectangles represent exons, black lines introns, gray areas represent UTR regions, red lines region deleted and purple, green and blue rectangles the protein domains. PAM and PINT are subdomains of the PCI domain, B) Micrographs of SYP-1 (red or grey scaled) and DAPI (blue) stained wild-type and *csn-5* mutants nuclei representing the various stages of the *C. elegans* gonad. Images are projections through half of a three-dimensional data stacks. Scale bar is 2 µm. PMT = pre-meiotic tip, TZ = transition zone, EP = early pachytene, MP = mid pachytene, LP = late pachytene. SYP-1 aggregates appear in the TZ-like stage of the gonad and persist through the LP-like stage. C) Whole gonad from wild-type and *csn* mutants SYP-1 and DAPI stained. Images show smaller gonads in *csn* mutants and lack of oocytes progressing through diakinesis. Scale Bar 16 µm. SYP-1 (grayscale) staining only of gonads showing aggregation throughout the gonad, starting at transition zone.

Three deletion alleles were analyzed in this study: *csn-2(tm2823)*, *csn-5(ok1064)* and *csn-6(ok1604)* (See also Materials and Methods). The *csn-2(tm2823)* allele is missing most of exon 3 which results in deletion of 28% of the coding region, including the PAM sub-domain [53] in the PCI domain (Figure 1A). The *csn-5(ok1064)* allele is missing exons 1, 2, and 3 which results in deletion of 64% of the coding region (including the MPN catalytic domain, Figure 1A). The *csn-6(ok1064)* allele is missing most of exons 1 and all of exon 2 which results in deletion of 43% of the coding region (including most of the MPN domain, Figure 1A). The *csn-2(tm2823)*, *csn-5(ok1064)* and *csn-6(ok1604)* alleles will be referred here collectively as *csn* mutants.

In wild-type nuclei, SC assembly is initiated at the transition zone (leptotene/zygotene) when SC proteins load on chromosomes (Figure 1B); the SC is fully assembled at pachytene. SC disassembly is initiated at the end of pachytene and CR disassembly is complete by the end of diakinesis. To determine if SC morphogenesis was affected in *csn* mutants, we performed immunohistochemical analyses using antibodies against SYP-1, SYP-4, HIM-3, and HTP-3 [5], [8], [12], [22] (Figure 1B,C; Figure S1 and S2). In all *csn* mutants, we observed smaller, morphologically different gonads compared to wild-type (Figure 1C), as previously published for *csn-5(RNAi)* [51], [52]. The nuclei in the *csn* mutant gonads were unevenly spaced throughout the gonad. There also appeared to be no distinct rachis (central canal) as in wild-type worms. The chromosomes of *csn* mutants clustered to one side of the nucleus (a polarized organization) as found in the wild-type transition zone (leptotene/zygotene) nuclei; this is indicative of meiotic entry [54]. Unlike wild-type, in the *csn* mutants these polarized nuclei were found throughout the gonad intermixed with nuclei with a dispersed chromosomal organization. The persistence of polarized chromosomes has been observed previously in synapsis defective mutants [11]. In addition to the persistent polarized chromosome organization, we also determined the mitotic/meiotic boundary using antibodies for lateral element proteins, HIM-3 and HTP-3. Since these lateral element proteins localize to chromosomes axes upon the transition from mitosis to meiosis. This localization occurred concurrently with polarization of chromosomal organization and did not show any defects in the germline of *csn* mutants. These data indicate: 1) the transition from mitosis to meiosis took place in the *csn* mutants, and 2) the localization of lateral element proteins of the SC was not perturbed in *csn* mutants (Figure S1). Thus, although gonads are smaller in *csn* mutants and have fewer nuclei, meiotic entry has occurred and SC assembly has initiated.

In contrast to the pattern of localization of lateral element proteins in the *csn* mutants, the CR protein SYP-1 showed an aberrant pattern of localization. SYP-1 protein aggregates (PC-like structures) were found in the transition-like zone at the distal end of the gonad and through the late-pachytene-like zone in the proximal end of the gonad. These occurred in 100% of the gonads examined (wild-type n = 26, *csn-2* n = 37, *csn-5* n = 34, *csn-6* n = 35; p<0.0005; Fisher's Exact Test) for all *csn* mutants (Figure 1B). CR/SYP aggregates were 4 fold wider than a typical SC (width of wild-type SC- 0.22 µm±0.23, n = 25, width of SC aggregate- *csn-2* 0.83 µm±0.23, n = 70 and *csn-5* 0.86 µm ±0.31, n = 90 p≪0.001 Mann Whitney Test) and typically there was one aggregate per nucleus (*csn-2* 1.12, n = 62 and *csn-5* 1.13, n = 82). While some nuclei contained a single SYP-1 aggregate with no additional SYP localization, most nuclei contained partially assembled linear SC (similar to that observed in wild-type gonads) in addition to the aggregate (detailed analysis below). As there are currently four SYP proteins, we examined the localization of SYP-4 in *csn-5* mutants as well as GFP::SYP-3 in *csn-2* and *csn-5* mutants to identify if the aggregation defects were specific to SYP-1 or generally affect all the CR components. SYP-3 and SYP-4 also form persistent aggregates suggesting the defects observed in the *csn* mutants are not specific to SYP-1 (Figure S2).

P-granules are germline RNA storage compartments that are composed of mRNAs and proteins; these include GLH-1 and PGL-1 proteins that are important for P-granule function. A recent paper by Bilgir *et al.*, 2013 described failure in SC assembly in *pgl-1* mutants. Since GLH-1 is known to be regulated by CSN-5 [55], SYP aggregation could be induced by a reduction in function of GLH-1 (and the consequent P-granule defects). CSN-5 promotes GLH-1 stabilization by competing with KGB-1 for binding to GLH-1 [51], [52]. If CSN-5 influenced SC through its role in P-granule function, than *glh-1* mutants should show similar phenotypes (SC aggregation) to *csn-5* mutants and *kgb-1* should destabilize SC (lack of SC). We did not observe any changes in SC structure, or any aggregation, in *kgb-1* and *glh-1* mutants (Figure S3.) We conclude from this, that P-granule destabilization is likely not the cause of SYP aggregation in *csn* mutants.

### *csn-2*, *csn-5* and *csn-6* mutants affect CR assembly

**Figure 2:**
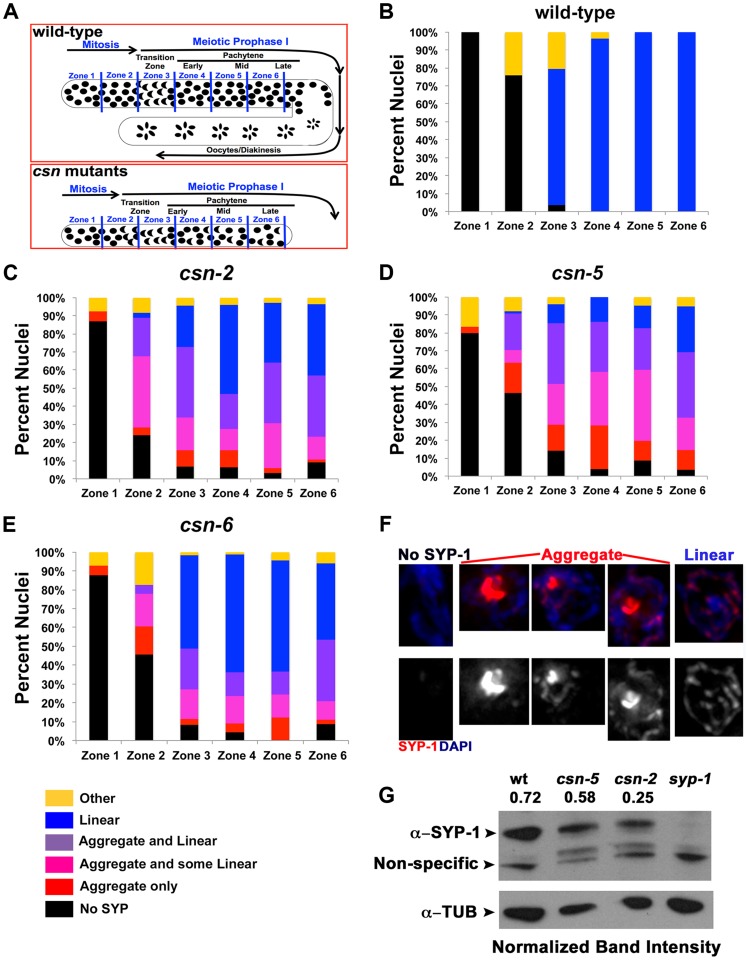
Quantification of the SYP-1 aggregates. A) Schematic representation of the zones of the *C. elegans* gonad. PMT = pre-meiotic tip, TZ = transition zone, EP = early pachytene, MP = mid pachytene, LP = late pachytene. B–E) Quantification of SYP-1 aggregates in zones of the gonad. Percent of nuclei with: no SYP-1 (black), linear SYP-1 (blue), aggregated SYP-1 (purple pink and red) and other (yellow), zones as in A, n nuclei scored wild type: 1123, *csn-2*: 868, *csn-5*: 1020, *csn-6*: 85, p<0.0005 for pairwise comparisons; Fisher's Exact Test F) Representative images of nuclei scored in C–D all taken from the same gonad in late pachytene of *csn-2* mutants, G) Western Blot confirming the reduction of expression of SYP-1 in *csn* mutants. Normalization values (α-SYP-1/α-TUB) shown are the average of 2 different experiments. Normalized intensities: wild-type 0.72±0.26, *csn-2* 0.24±0.20 and *csn-5* 0.58±0.11.

Having determined all three *csn* mutants display SYP aggregation, we asked if the defects in SC assembly were indistinguishable in our mutants. Not all nuclei within the transition-like zone and pachytene-like zone had aggregates. The gonads were divided into six zones sized as in Colaiacovo 2003 ([4], Figure 2A) and were scored for the percent of nuclei with aggregates in each zone. Each zone represents a size unit (36 µM×36 µM window) organized sequentially (zone 1 being the distal pre-meiotic tips (PMT) and zone 6 the proximal late pachytene region). This division into zones was performed according to the standard protocol for quantitative analysis of early to mid-meiotic events in the *C. elegans* germline, (*e.g.*, RAD-51 analysis, also see Materials and Methods). We divided the SYP-1 localization pattern to 6 categories and quantified the percent of nuclei in each category in each zone. Linear refers to SYP-1 that is morphologically similar to that observed in wild-type. Aggregated SYP was divided into three categories reflecting the amount of linear SYP-1 that is present in nuclei alongside with aggregate: linear (most of the DAPI had linear SYP-1), aggregate only (no linear SYP-1) and intermediate (some linear). *csn-2* and *csn-6* mutants showed a lower percent of nuclei with SYP-1 aggregates compared to *csn-5* (Figure 2B–D percent nuclei with aggregates out of total number of nuclei; wild-type 0%, *csn-2* 41%, *csn-5*, 57%, *csn-6* 30%, for statistics and n values see figure legend). Analysis of SYP-1 localization in the *csn-2; csn-5* double mutant revealed similar findings; however early meiotic nuclei tended to have low aggregation levels, comparable to *csn-2*, while later meiotic nuclei were more similar to *csn-5* (Figure S4). Overall, the percent of aggregated nuclei varied between gonads (*e.g.*, 50–72% for *csn-5*), but mutant gonads always contained SYP-1 aggregates and wild-type gonads never harbored SYP-1 aggregates. The early appearance of SYP aggregates as SC assembly initiates (zone 2–3) indicates that the primary defect observed in *csn* mutants is in SC assembly.

SYP-1 aggregation could result from over-expressing SYPs [12]. To address this point; we performed a Western blot analysis to determine the level of SYP-1 in the *csn-2* and *csn-5* mutants. Both *csn* mutants had a reduced level of SYP-1 compared to wild-type (Figure 2G, *csn-2* 40%±23 and *csn-5* 80%±5 of wild-type, average between experiments). We also performed a similar experiment using HTP-3 as a normalization control and found similar results (Figure S5 *csn-2* 76%±15 and *csn-5* 65%±6 of wild-type). We also utilized a cytology-based assay to quantify the amount of nuclear SYP-1 protein in *csn* nuclei compared to wild-type. In this analysis, the intensity of an image was used for calculating the amount of protein using standard methodologies (for details see Materials and Methods). We observed a decrease in nuclear SYP-1 in the *csn-5* mutant, but not for *csn-2*, compared to wild-type (Figure S5). These data argue that the SYP-1 aggregates are not the result of detectable over-expression of SYP-1.

### *csn-2* and *csn-5* are required for gonad proliferation and fertility

**Figure 3:**
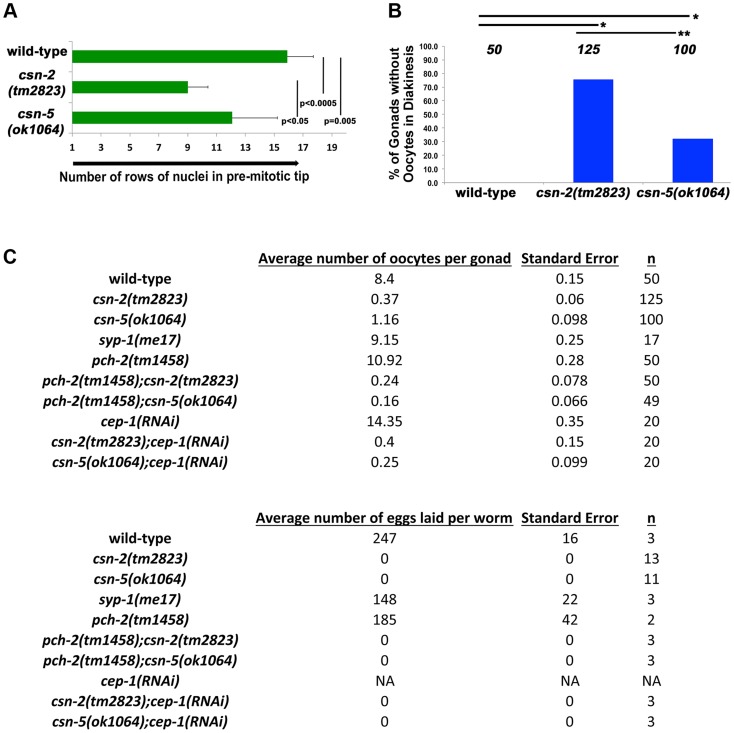
Quantification of the lack of oocytes and fecundity test. A) Relative sizes of the pre-meiotic tips for wild-type and the *csn* mutants. The size of the mitotic zone is reduced in *csn* mutants. n = 10 for each strain p<0.0005 for wild-type vs. *csn-2*; p = 0.005 for wild-type vs. *csn-5* and p<0.05 for *csn-2* vs. *csn-5*; Mann Whitney Test B) Quantification of the number of gonads that contained oocytes in diakinesis for the *csn* mutants, *p_MW_<0.0005 and **p_MW_<0.005, Mann Whitney Test C) Top: the average number of oocytes in diakinesis for the *csn* mutants and the *csn* mutant, apoptosis checkpoint double mutants. Bottom: the average number of eggs laid for *csn* mutants and apoptosis checkpoint double mutants. *csn* mutants have a severe reduction in the number of oocytes and lay no eggs.

The overall length of the gonads of *csn* mutants is shorter than observed in wild-type (Table S4), which could result from reduced proliferation of mitotic cells. If mitotic proliferation (prior to meiotic entry) was affected, the size of the mitotic zone (*i.e.*, the PMT) would be shorter in *csn* mutants. As nuclei enter meiosis, they acquire a polarized configuration of chromosomes indicating meiosis was initiated. We used this polarization to measure the length of the PMT of gonads for each genotype. Our analysis focused on *csn-2* and *csn-5*, although *csn-6* showed similar phenotypes that were not quantified in such detail. In the *csn* mutants, the PMT region was 60% and 77% of the length in the wild type (Figure 3A). Similar analysis using HTP-3 antibody as a marker for meiotic entry revealed similar findings: PMT region was ∼60% shorter than in wild-type for both mutants (for each strain p = 0.004 for wild-type vs. *csn-2*; p = 0.002 for wild-type vs. *csn-5* and p = 0.83 for *csn-2* vs *csn-5*; Mann Whitney Test) One interpretation of these data is that the transition from mitosis to meiosis occurs earlier in these mutants compared to wild-type.

When nuclei move to diakinesis, the final stage of meiotic prophase I, wild-type gonads contain an average of 8.1±1.1 diakinesis nuclei. These diakinesis nuclei are also referred to as oocytes [56], although the cellularization process occurs only towards the end of diakinesis. Unlike wild-type, most gonads of *csn* homozygotes lacked diakinesis nuclei/oocytes (Figure 3B). In *csn-2* mutants, only about 25% of the gonads had diakinesis nuclei, and an average of 0.8±0.76 per gonad (Figure 3C). In *csn-5* mutants, 65% of the gonads had diakinesis nuclei with an average of 1.23±0.98 per gonad. We performed an egg lay assay to determine the number of eggs laid and their viability. For the *csn* mutants, no eggs were laid in a three-day period; in contrast wild-type worms had an average of 247±16 eggs laid per worm (Figure 3C).

### *csn-2*, *csn-5* and *csn-6* are required for pairing stabilization

**Figure 4:**
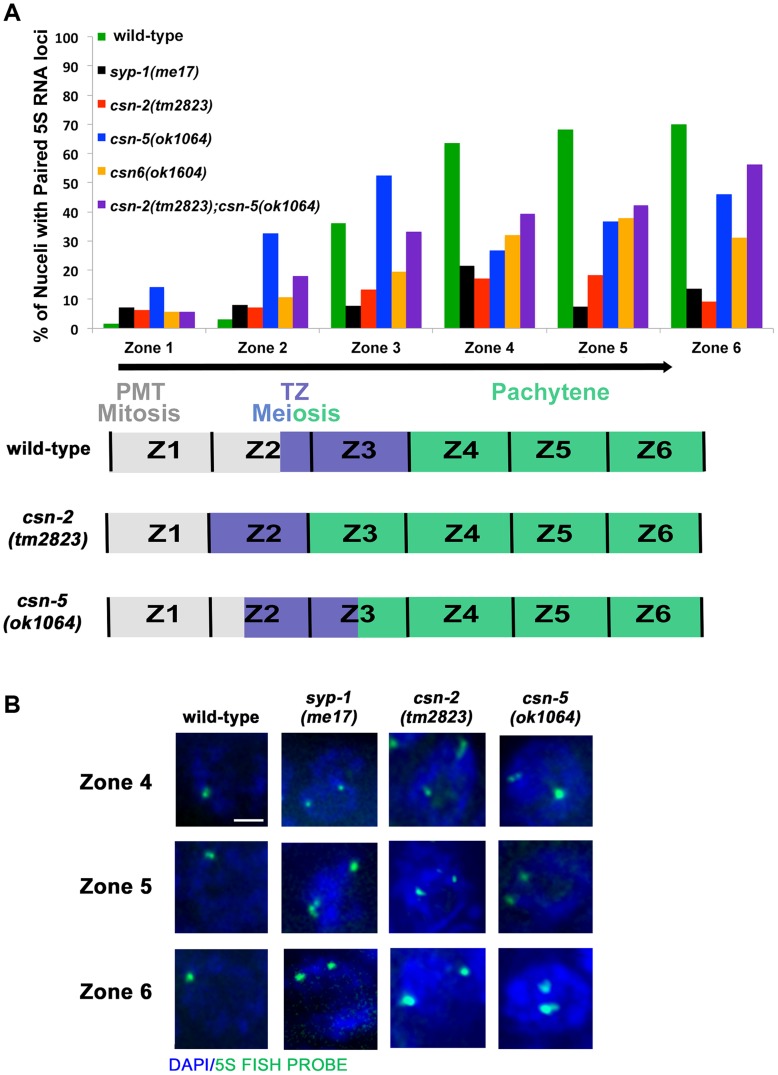
Pairing stabilization is affected in *csn* mutants. A) Analysis of pairing stabilization between wild-type, *syp-1(me17)*, *csn* mutants. A schematic representation of the timing of meiotic stages relative to the zones in the *C elegans* gonad. zone 1 = pre-meiotic tip, zone 2 and 3 = transition from mitosis to meiosis, zone 4–6 = pachytene. The black arrow represents the movement of nuclei through the stages (zones) of meiosis. *csn* mutants show defects in pairing stabilization number of nuclei counted and *p*-values can be found in Sup.Tables 1 and 2. B) High magnification micrographs of individual nuclei. Images are projections through three-dimensional data stacks. 5S FISH probe foci are in green and DAPI stained chromosomes are in blue. zone 4 = early pachytene, zone 5 = mid pachytene, zone 6 = late pachytene. Scale bar is 2 µm.

Pairing interactions between homologous chromosomes are initiated in a SC-independent manner at specific chromosomal sites (pairing centers). The term pairing stabilization describes pairing interactions that spread outside the pairing centers and lead to the persistence of homolog association throughout pachytene. In *syp* mutants, loci distant from the pairing centers exhibit a very low level of pairing throughout meiosis [54]. Since the data indicated that *csn* mutants lack a fully functional SC; we expected to find that pairing stabilization had been compromised in the *csn* mutants, similar to *syp* mutants. To test this, we used a 5S ribosomal RNA locus on the center of chromosome V to analyze pairing interactions between homologous chromosomes by fluorescence *in situ* hybridization (FISH). The gonads were divided into six zones and were scored for the percent of nuclei with paired 5S loci in each zone ([57], Figure 4 and Tables S1 and S2 for n values and statistics).

In zone 1, as expected, wild-type and *syp-1(me17)* control nuclei, as well as the *csn* mutants, showed little to no homologous pairing, fewer than 15% of 5S loci had paired chromosomes. As the nuclei progressed through meiotic prophase I, wild-type chromosomes initiated pairing and maintained high levels of pairing through the pachytene zones. In *syp-1(me17)* because there is no SC formed, mutant chromosomes remained mostly unpaired throughout the germline (Figures 4A and 4B). Pairing levels in *csn-2* mutants never exceeded 20% of the 5S loci paired in any zone, indicating the majority of the chromosomes were unpaired. Overall, *csn-2* and *syp-1(me17)* mutants exhibited similar pairing defects throughout meiotic prophase I (Figure 4A and 4B, Table S1 for n values and S2 for statistics). In contrast to *csn-2* mutants, *csn-5* mutants initiated pairing similarly to wild-type. Since the transition from mitosis to meiosis is occurring earlier in *csn-5* mutants, pairing initiated at zone 2, compared to zone 3 in wild-type (Figure 4A). In zone 4, *csn-5* mutants showed a reduction in the percent of nuclei with paired chromosomes, but the levels were intermediate between those observed in wild-type and *syp-1* or *csn-2* mutants. The percent of nuclei with paired chromosomes for the *csn-5* mutant remained higher than the *csn-2* mutant for zones 5 and 6. *csn-6* showed an intermediate phenotype with low pairing levels as meiosis initiated (similar to *csn-2*) that then increased (similarly to *csn-5*), but never exceeded wild-type pairing levels. Meiotic nuclei of *csn-2; csn-5* double mutants tended to have low pairing levels overall: the pairing levels in late pachytene nuclei were similar to *csn-5* and statistically different from *csn-2*. When taken together these findings are consistent with a view where defects in SC assembly perturb pairing stabilization. The percent of nuclei with linear SYP-1 (Figure 2B–E) frequently exceeded the percent of nuclei paired. Therefore these data suggest that SYP assembled in a linear manner on chromosomes in *csn* mutants cannot support pairing stabilization. This SC-like localization (linear SYP-1) likely represents non-homologous synapsis and/or SYP assembly between sisters.

### Meiotic recombination and crossover formation are perturbed in *csn* mutants

**Figure 5:**
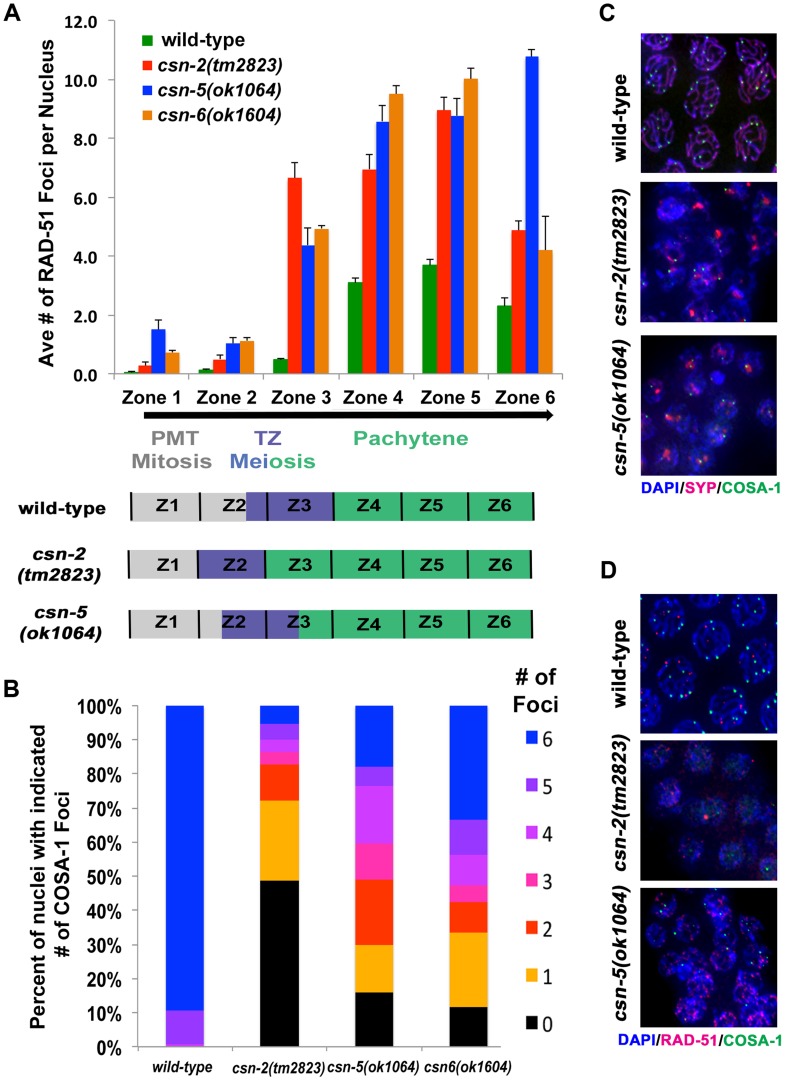
Accumulation of recombination intermediates and reduced crossover formation in *csn* mutants. A) Analysis of RAD-51 foci in wild-type compared to *csn* mutants. Position along the x-axis refers to the zone in the gonad (Figure 4). RAD-51 foci accumulate upon entrance to meiosis in *csn* mutants. Numbers of nuclei counted and *p*-values can be found in Sup. Table 3. Schematic representation of the timing of meiotic stages relative to zones scored in the *csn* mutants compared to wild-type. B) Quantitative analysis of COSA-1 foci in late pachytene of the wild-type and zone 6-like section of the *csn* mutants color code for number on COSA-1 is at right. Number of designated crossovers marked by COSA-1 is reduced. Number of nuclei scored: wild-type n = 123, *csn-2* n = 111, *csn-5* n = 94, *csn-6* n = 78 p<0.0005 for comparison between wild-type and mutants and *csn-2* to *csn-5* or *csn-6*, p = 0.13 for *csn-5* to *csn-6*, Mann Whitney Test, C) Micrograph images of COSA-1 foci (green), chromosomes DAPI (blue), and SYP-1 (red) in wild-type and *csn* mutants. D) Micrograph images of RAD-51 foci (red), COSA-1 foci (green), and chromosomes DAPI (blue) in wild-type and *csn* mutants. Images are projections through three-dimensional data stacks. Scale bar is 2 µm.

In mutants that do not form the SC, events downstream of pairing and SC assembly such as meiotic recombination are perturbed [54]. We expected the *csn* mutations would have a similar effect on recombination. RAD-51 is a strand exchange protein used as an indirect marker for DSB formation and subsequent repair in *C. elegans* [4]. The gonads were divided into zones as previously described and the numbers of RAD-51 foci per nucleus were counted (Figure 5A and Table S3 for n values and statistics).

Mitotic nuclei in zones 1 and 2 showed very low levels of RAD-51 both in wild-type and *csn-2* mutants. *csn-5* and *csn-6* mutants exhibited slightly increased levels of RAD-51 foci in mitotic nuclei. RAD-51 foci levels increased at the entrance to meiosis in all genotypes tested, as expected from the induction of meiotic DSBs. The increase in RAD-51 foci/nucleus occurred earlier in *csn* mutants, likely due to the fact meiotic entry occurred earlier. Despite the similarity of RAD-51 localization patterns in the distal part of the germline, the overall levels of RAD-51 foci in early prophase were about 2-fold increased in *csn* mutants compared to wild-type. The levels of RAD-51 foci in the *csn* mutants remained higher than wild-type in zones 5–6, indicating the repair of DSBs was affected. In late pachytene, we observed a difference between *csn-2* and *csn-6* vs. *csn-5* mutants: *csn-5* mutants maintained RAD-51 foci at high levels, while they decreased in *csn-2* and *csn-6* mutants.

In *C. elegans*, one obligatory crossover is observed per chromosome pair [4]. COSA-1, a conserved cyclin related protein, localizes to crossovers and can be used to monitor the number of designated crossovers per nucleus (normally six, one for each wild-type bivalent formed) (Figure 5C and D). A reduction in COSA-1 foci suggests a defect in crossover formation. We tested the *csn* mutants using a GFP-tagged COSA-1 [58] to determine if crossover formation was affected. COSA-1 foci were measured at the last zone of late pachytene (as in [58]). Wild-type nuclei had an average of 5.8±0.04 COSA-1 foci (10% of nuclei with less than 6 foci), indicating designated crossovers had been properly formed (Figure 5C, see legend for statistics and n values). However, in the *csn-2* mutant, 95% of the nuclei had less than six foci (1±0.16 foci per nucleus). In the *csn-5* mutant, we observed a wider distribution of the number of COSA-1 foci observed, with 82% of the nuclei with less than six foci (2.8±0.21 foci per nucleus,). In the *csn-6* mutant, we observed similar distribution to that of *csn-5*, with 67% of the nuclei with less than six foci (an average of 3.4±0.23 foci per nucleus). The average numbers of COSA-1 foci were significantly different between the *csn-2* and *csn-5* or *csn-6* mutants. These data suggest a role for CSN/COP9 in crossover formation.

### Apoptosis is increased in *csn-2* and *csn-5* mutants and dpMPK-1 levels are reduced

In synapsis-defective mutants lack of synapsis [59], as well as an accumulation of DSBs [4] results in increased apoptosis at late pachytene [54]. CED-1, is expressed during the process of engulfment; a mechanism of clearing apoptotic corpses from the germline in late pachytene. Thus, the fusion protein *ced-1*::GFP, exclusively surrounds apoptotic nuclei and is used as a marker to detect apoptosis.

**Figure 6:**
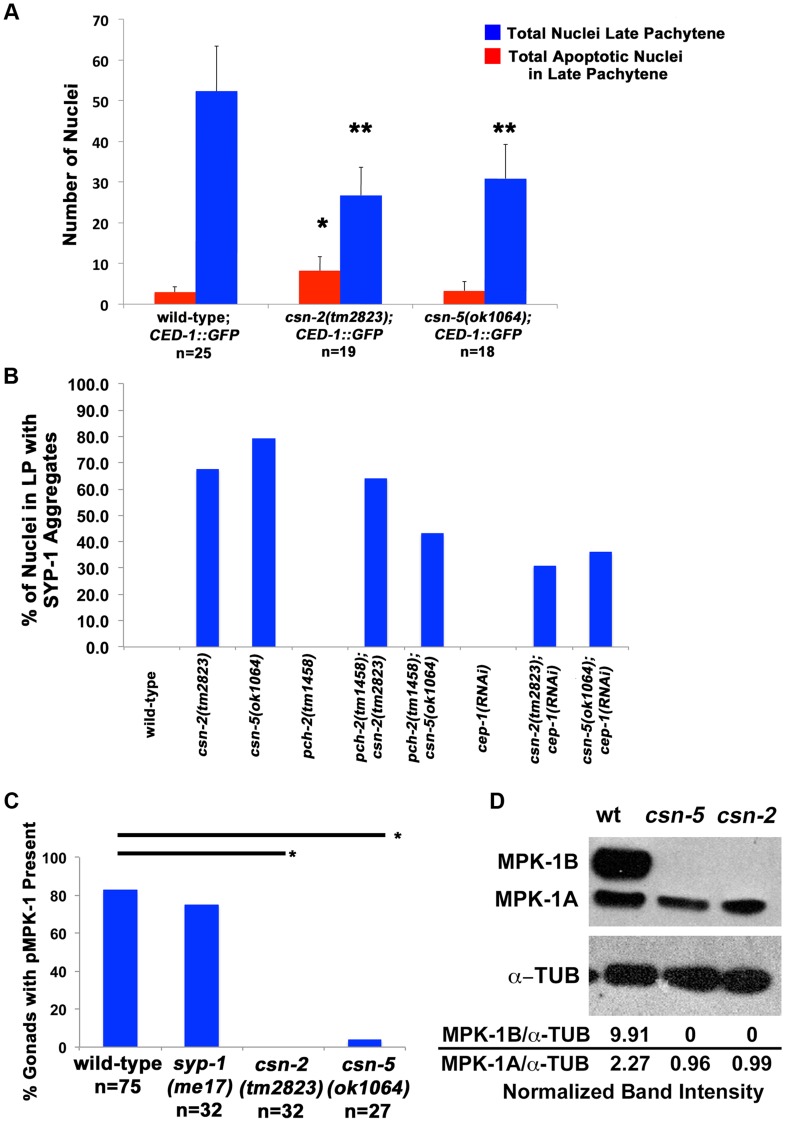
Apoptosis and MPK-1 expression are altered in *csn* mutants. A) Quantification of the number of nuclei with CED-1::GFP present in late pachytene of the *C. elegans* gonad. Red bars represent the total number of apoptotic nuclei in the late pachytene region. The blue bars represent the total number of nuclei in the late pachytene region. Apoptosis is increased in *csn-2* mutants, but not in *csn-5* mutants, *p_MW_<0.0005. There is also a reduction of overall nuclei in the late pachytene region of the gonad in both *csn* mutants, **p_MW_<0.0005. wild-type n = 25 gonads; *csn-2* n = 19; and *csn-5*, n = 18, B) Analysis of SYP-1 aggregate phenotype in *csn* mutants and apoptosis checkpoint double mutants. Bypassing the apoptotic checkpoints reduces the number of nuclei with aggregates. Total number of nuclei counted and *p*-values can be found in Sup. Table 5. C) Quantification of dpMPK-1 expression via IF analyses. *csn* mutants lack MPK-1 staining in late pachytene and in diakinesis, *p_FET_<0.0005. wild-type n = 75, *csn-2* n = 32, *csn-5*, n = 27, *syp-1(me17)* n = 32, D) Western Blot confirming the lack of expression of MPK-1B in *csn* mutants. MPK-1A is mostly somatic and MPK-1B is germline specific. Normalization values (α-MPK-1/α-TUB) shown are the average of 3 different experiments. Normalized intensities: wild-type 2.27±1.03, *csn-2* 0.96±0.42 and *csn-5* 0.99±0.09.

Both *csn-2* and *csn-5* mutants had lower average numbers of nuclei in late pachytene (Figure 6A wild-type average = 52.2; *csn-2* = 26.7; and *csn-5* = 30.9). However, only *csn-2* had a significant increase in apoptosis (4-fold) while *csn-5* had apoptotic levels similar to wild-type (Figure 6A wild-type average = 2.96; *csn-2* = 8.2; and *csn-5* = 3.3 apoptosis levels were not examined in *csn-6* mutants). When normalized for the number of nuclei in late pachytene, both mutants showed increased apoptosis, and, as expected, *csn-2* mutants showed a larger increase. This is the more appropriate analysis since *csn* mutants have less germline nuclei.

There are two apoptotic checkpoints in *C. elegans* meiosis that are activated by unsynapsed chromosomes: the synapsis checkpoint mediated by PCH-2 [59] and the meiotic recombination checkpoint mediated by CEP-1/p53 [60]. We investigated whether removing these two genes could bypass the DNA damage/synapsis checkpoint leading to apoptosis in the *csn* mutants. *pch-2(tm1458); csn-2(tm2823)* and *pch-2(tm1458); csn-5(ok1064)* double mutants were generated and *cep-1(RNAi)* was performed on the *csn* mutants. Overall gonad length, number of oocytes in diakinesis, and the number of nuclei containing aggregates were measured. If increased apoptosis in late pachytene was the reason for the severe reductions in oocyte numbers, in *csn* mutants, then bypassing the checkpoint function should increase the numbers of diakinesis nuclei and increase the overall size of the gonad. We observed no change in overall gonad length (Table S4) between the single *csn* mutants and the corresponding double mutants, nor any increase in oocytes in diakinesis in young adults (one day post-L4, Figure 3C). Since clearing apoptotic corpses may be slow, we scored the same genotypes two days later, giving the opportunity for accumulation of cells (in double mutants) otherwise destined for apoptosis (*csn* single mutants). In *csn-5* mutants overall gonad length decreased with age, possibly due to the defects in mitotic proliferation (less nuclei there are, the gonad gets shorter). However, both *csn-5; pch-2* and *csn-5; cep-1* mutants showed increased gonadal size compared to *csn-5* single mutants (almost double the size, table S4). These data indicates that both the synapsis and the DNA damage checkpoint are activated in *csn-5* mutants and are clearing nuclei through apoptosis.

Next, we assayed how removing the DNA damage and synapsis checkpoints would affect SC morphology in *csn* mutants. For this analysis we scored two categories: nuclei with linear SYP-1 localization and nuclei with aggregates (with our without other forms of SC). We measured the percent of nuclei with aggregates in double mutants with perturbed apoptotic machinery compared to single mutants. *csn-5(ok1064); pch-2(tm1458), csn-2(tm2823); cep-1(RNAi), and csn-5(ok1064); cep-1(RNAi)* double mutants exhibited a decrease (2-fold) in the number of nuclei with SYP-1 aggregates in late pachytene compared to the respective single *csn* mutant (Figure 6B and Table S5 for statistics). Therefore, nuclei with linear SYP were more frequently found in double mutants (in which the checkpoints are removed), indicating that functional checkpoints are associated with reduction in nuclei with linear SYP and promoting the aggregation of SYP proteins.

Given the known physical interaction between CSN-5 and MPK-1, a MAPK signaling protein [61], [62], and the substantial pachytene arrest (defects in progression from pachytene to diplotene) observed in *csn* mutants, we examined whether MAPK signaling was disrupted in *csn* mutants. Phosphorylated MPK-1 (dpMPK-1), the active form of MPK-1, is found in two distinct regions of the germline: late pachytene and late diakinesis. Nuclei of *mpk-1* null mutants completely arrested at mid-pachytene and no oocytes were observed [63]. However, when only MPK-1 phosphorylation is eliminated (*let-60* mutants) limited pachytene arrest occurred and oocyte numbers were severally reduced [64]. The increase of dpMPK-1 pachytene serves as a signal for pachytene progression. The diakinesis dpMPK-1 is required for maturation of oocytes [65], [66]. Using an antibody for phosphorylated MPK-1, we stained wild-type and *csn* mutant gonads. None of the *csn-2* mutants examined had dpMPK-1 staining in late pachytene while only 3% of the *csn-5* mutants had dpMPK-1 staining. In contrast, 82% of wild-type and 75% of *syp-1(me17)* gonads had dpMPK-1 staining (Figure 6C). MPK-1 has two isoforms in *C. elegans*, MPK-1A (43.1 kD) which is mostly somatic and MPK-1B (50.6 kD) is germline specific [60], [63]. We quantified the intensity of the bands and normalized to the tubulin controls (Figure 6D). In wild-type, both isoforms were detected with MPK-1A having an average normalized intensity of 2.27. The MPK-1A band was detected in both *csn* mutants (*csn-2* = 0.99 and *csn-5* = 0.96 normalized intensities), although it was 2-fold lower in both mutants. The germline MPK-1B had an intensity of 9.91, but was not detected in either *csn* mutant (Figure 6D). These data indicate that *csn* mutants lead to reduced MAPK/MPK-1 signaling which almost completely blocks pachytene exit and severely reduces oocyte numbers. This data is consistent with the observation that removing apoptosis checkpoints (*pch-2* or *cep-1*) could not increase oocyte numbers in *csn* mutants: even if more nuclei survived apoptosis, they could still not exit pachytene arrest in the absence of dpMPK-1.

### Decreasing neddylation and ubiquitination modify the phenotypes observed in *csn* mutants

**Figure 7:**
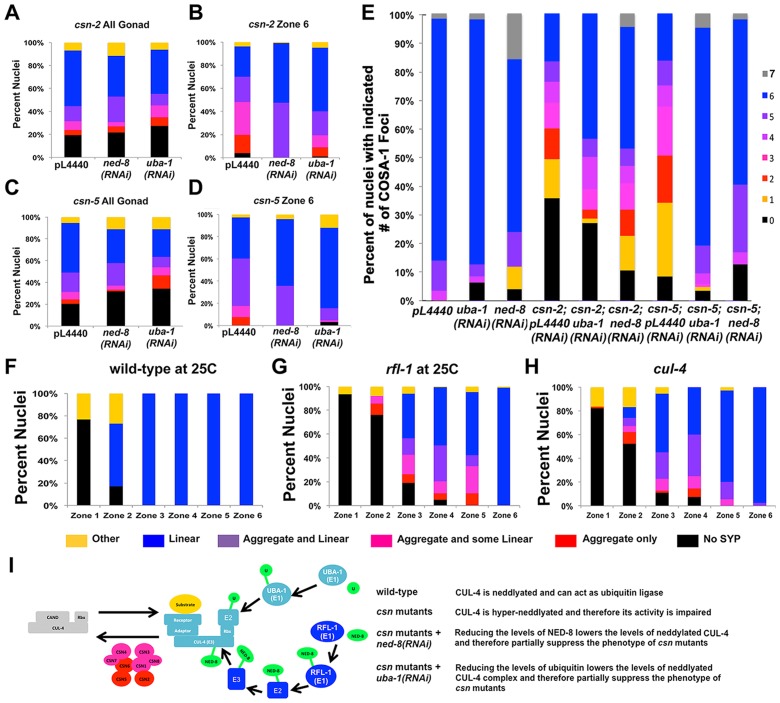
*csn* mutant genetically interact with the ubiquitination-neddylation pathway in the regulation of SC assembly and recombination. A–D) Quantification of SYP-1 aggregates. A and C are data from all gonad, while B and D is from late pachytene nuclei. Percent of nuclei with: no SYP-1 (black), linear SYP-1 (blue), aggregated SYP-1 (purple pink and red) and other (yellow), zones as in Figure 2A, n nuclei scored for whole gonad *csn-2*: with pL4440 = 2023 with *ned-8(RNAi)* = 430, with *uba-1(RNAi)* = 1121, *csn-5*: with pL4440 = 2096, with *ned-8(RNAi)* = 441, with *uba-1(RNAi)* = 1014. E) Quantitative analysis of COSA-1 foci in late pachytene wild-type: Percent of nuclei with: zero (black) one (orange), two (red), three (pink) four (magenta), five (purple), six (blue) and seven (gray), n nuclei scored for pL4440 = 57, with *ned-8(RNAi)* = 47, with *uba-1(RNAi)* = 25, *csn-2*: with pL4440 = 168, with *ned-8(RNAi)* = 66, with *uba-1(RNAi)* = 126, *csn-5*: with pL4440 = 152, with *ned-8(RNAi)* = 47 with, *uba-1(RNAi)* = 83, pL4440 = empty vector control vs. *ned-8(RNAi)* on *csn-2* or *csn-5* p = 0.002, p<0.001, Fisher's Exact Test, pL4440 vs. *uba-1(RNAi)* on *csn-2* or *csn-5* p<0.001 Mann Whitney Test). F–H) Quantification of SYP-1 aggregates in zones of the gonad for the indicated genotypes, as performed in figure, number of total nuclei scored: wild-type 25 n = 641, *rfl-1* at 25 n = 1674, *cul-4* n = 542 2, I) Schematic representation of the pathway examined in the experiment.

We have shown that synapsis (SC assembly) and recombination are perturbed in three *csn* mutants. The main role of the CSN/COP9 signalosome is in deneddylation of CRLs. Therefore, the *csn* mutant phenotypes could be attributed to the increased neddylation in the absence of a functional CSN/COP9 signalosome. The effect of complete absence of neddylation on the germline cannot be examined since null mutants in *ned-8*, the only gene encoding for the small modifying protein NED-8, are larval lethal. To test the hypothesis that over neddylation leads to the phenotypes described we have decreased the levels of neddylation via RNAi for *ned-8* in *csn-2* and *csn-5* mutants. As seen in Figure 7B and D, (detailed distribution in Figure S6 and statistics in Table S6), *ned-8(RNAi)* partially suppressed the synapsis defects of *csn* mutants (pL4440 = empty vector control vs. *ned-8(RNAi)* on *csn-2* p<0.001, on *csn-5* p = 0.002, Fisher's Exact Test). More strikingly the levels of designated crossover (COSA-1) of *csn* mutants were partially restored (Figure 7E). Since CSN/COP9 signalosome typically deneddylates CRLs, which are ubiquitin ligases, the *csn* mutant phenotypes could also potentially be suppressed by reducing ubiquitination levels. As with neddylation, null mutants in genes essential for the ubiquitination pathway die prior to the adult stage and cannot be used in our studies. We have used RNAi for the sole E1 ubiquitin ligase of *C. elegans*, UBA-1, to test the hypothesis that the phenotypes of *csn* mutants can be attributed to increased ubiquitination. As with neddylation, *uba-1(RNAi)* partially suppressed both the defects in SC assembly (Figure 7 B and D) and the crossover defects of *csn* mutants (Figure 7E). If imbalance of neddylation is the cause of the phenotype observed in *csn* mutants, hyper-neddylation (*csn* mutants) and hypo-neddylation (mutants in the NED-8 pathway) will lead to similar phenotypes. The *ned-8(RNAi)* is weak; it does not lead to increased lethality although the null allele has a lethal phenotype. Therefore, it is not surprising that *ned-8(RNAi)* on wild-type did not lead to any phenotype. However, examination of an E1 NED-8 ligase (*rfl-1*) revealed defects in SC assembly reminiscent of the *csn* mutant phenotype (Figure 7G), defects not observed in the control (Figure 7F). Finally, we sought to identify the E3 CULLIN ligase, which is the target of CSN/COP9 signalosome. This analysis is limited because many mutants of *cul* genes are embryonic or larval lethal. We have identified one *cul-4* mutant allele (a C-terminal truncation) that exhibited mild defects in SC assembly, including aggregation of SYP-1 (Figure 7H). CUL-4 is therefore, a possible CSN/COP9 signalosome target. This genetic analysis is consistent with a canonical role for CSN/COP9 signalosome in the CRL pathway: regulating CUL-4 via denaddylation and keeping the balance between neddylation and denaddylation.

Taken together, these data indicate the CSN/COP9 signalosome has multiple roles in meiosis: the signalosome affects the number of germline nuclei, SC assembly and stabilization, recombination, MAPK signaling and promoting pachytene exit.

## Discussion

### CSN/COP9 is required for chromosome synapsis, pairing and recombination during *C. elegans* meiosis

The CSN/COP9 signalosome has diverse and well-documented somatic functions, yet the understanding of its role in meiosis is limited [28]. Studies of the *C. elegans* and *D. melanogaster* CSN/COP9 indicate it plays a critical role in the regulation of Vasa/P-granule proteins in the germline [42], [55], [67]. Here, we show CSN/COP9 has a previously unknown meiotic function: it is essential for proper SC assembly, independent of its P-granule role in the germline. We demonstrate that events following SC assembly (*e.g.*, stabilization of homolog pairing interactions and the repair of meiotic DSBs) are perturbed as well. The three *csn* mutants show similar, but not identical effects on these processes; in the absence of CSN/COP9, the SYPs (CR proteins) aggregate. In *C. elegans*, stabilization of pairing interactions is absolutely dependent on SC formation and independent of DSB formation and repair [57]. Therefore, it is reasonable to propose that the pairing defects observed in CSN/COP9 mutants stem from defects in SC formation. The limited amount of SC that assembles on chromosomes in *csn* mutants cannot support wild-type levels of stabilization of pairing interactions (by FISH analysis). In the absence of fully stabilized pairing interactions, unresolved recombination intermediates (marked by RAD-51) accumulate. This leads to a reduction in the numbers of designated crossovers (marked by COSA-1 foci) in *csn* mutants and an elevation of apoptosis. The magnitude of these phenotypes in *csn-2* mutants closely resembles that of *syp* null mutants, supporting our model that the later meiotic defects (pairing and recombination) stem from an inability to form functional SC. The exact magnitude of the effects on recombination and pairing initiation is different between the mutants and may point to additional roles of components of the CSN/COP9 signalosome in pairing and recombination (more discussion below). Importantly, all three *csn* mutants we have examined affect synapsis, pairing, and recombination and therefore we are confident in our claim for a role for the CSN/COP9 complex in these key meiotic events.

### CSN/COP9 is required for normal levels of germline proliferation, MPK-1 activation and pachytene exit

Consistent with previous studies in *C. elegans* utilizing RNAi [51], [52] the three *csn* mutants have a reduced gonad size. Our data suggest this reduction is due to a proliferation defect, as the number of mitotically dividing nuclei in the pre-meiotic tip is reduced in the *csn* mutants. *Drosophila csn4* and *csn5* mutants cannot stabilize Cyclin E leading to defects in cell cycle progression of mitotically proliferating germline nuclei [46]. This suggests a conserved function of the CSN/COP9 signalosome in pre-meiotic germline proliferation.

Once nuclei of *csn* mutants enter meiosis, chromosomes cluster to one side of the nucleus as in wild-type; unlike wild-type however, a portion of these nuclei do not re-acquire the normal dispersed chromosomal organization as they progress through meiosis (Figure 1B and C). This phenotype of persistent polarized chromosome organization is reminiscent of *syp* null mutants during meiotic progression. This finding, together with the observation that *csn* mutants do initiate meiotic recombination and form some designated crossovers, is consistent with meiotic progression from leptotene to pachytene in these mutants. However, unlike syp mutants, *csn* mutants produce almost no diakinesis/oocyte nuclei. Reduced oocyte production would results from the decrease in numbers of germ cells, yet the effect on oocyte production is greater than expected from the reduction in number of pachytene nuclei destined to be oocytes. We have found MAPK signaling (dpMPK-1) is reduced in *csn* mutants. MAPK signaling is essential for pachytene progression [63] and so we infer that reduced dpMPK-1 levels are likely the primary contributor to the severe reduction in oocyte numbers in *csn* mutants. As synapsis defective mutants (*e.g.*, *syp-1*) still exit pachytene and form oocytes in comparable levels to wild-type, the lack of MAPK signaling in the *csn* mutants defines another function for the CSN/COP9 complex and is not a secondary effect of the synapsis defects. Since CSN-5 physically interacts with MPK-1 [61], [62], the absence of MPK-1 phosphorylation, may be due to the absence of this interaction. The role of the CSN/COP9 signalosome in pachytene exit seems to be conserved, as similar to our observation, *Drosophila csn8* and *csn4* mutants arrest at the pachytene-diplotene transition [68], [69].

### The relation between the linear and the aggregate forms of the CR proteins

In *C. elegans*, CR/SYPs assembly can be misregulated in certain meiotic mutants without forming aggregates [7], [9], [11], [14]. These aberrant forms of SC assembly appear to be fully formed SCs that are assembled in the wrong chromosomal context. The situation found in the *csn* mutants is different: in addition to aberrant SC assembly (short stretches) ∼50% of nuclei contain one SYP aggregate. In *C. elegans*, lack of any one of the four SC protein results in elimination of the other SYPs without their aggregation [13], indicating that mechanisms exist to remove SYPs not bound to DNA. *csn* mutants are therefore likely perturbed in mechanisms designed to clear aggregated SYPs, and assemble a “SC-like” structure which is invisible to the degradation machinery. In yeast, the CR protein is continually loaded on the SC, even in mid-prophase [70]. If a similar rapid exchange of SYPs occurs during *C. elegans* meiosis, SC assembly defects (problems in SC assembly upon meiotic entry) and SC stabilization defects (throughout pachytene) are related, due to continuous assembly of SC protein occurring throughout prophase. This may explain why a mutant that affects SC assembly (*csn-5*) was retrieved in an enhancer screen for a mutant showing SC disassembly defects.

Although it is likely that the formation of CR aggregates reflects an aberrant form of SC assembly, it is not clear if these aggregates are the problem or an attempt to solve a problem. In other words, it could be that the CRs which cannot be properly loaded onto chromosomes aggregates and therefore would not be available for SC formation. Alternatively, CRs may be loaded on to chromosomes, but then identified as aberrant CR, removed and then form aggregates which acts as reservoirs of CR proteins waiting to be re-loaded. Since linear (and non aggregated CR) seem to be a better predictor for failed meiosis, we tend to favor the hypothesis were CR aggregates are formed in response to attempts to correct an aberrantly formed SC. First, mutants with more linear SYP-1 (*csn-2* and *csn-6*) in early meiotic stages (zone 3) show more severe defects in pairing compared with a mutant with a higher percentage of SYP-1 aggregation (*csn-5*). Second, nuclei with linear SYP-1 are preferentially selected for elimination by apoptosis. Lastly, if aggregation is only an assembly defect, then the percent of aggregated nuclei should increase as meiosis progresses, which does not occur. This is more consistent with a model of SYP shuttling between an assembled and an aggregated from.

We have shown that the percent of nuclei undergoing apoptosis is increased in both *csn-2* and *csn-5* mutants. This active elimination of nuclei by apoptosis reduces the size of the germline over time (*csn-5; pch-2* gonads were longer compared to *csn-5* single mutant at day 3). Both the synapsis checkpoint and the DNA damage checkpoints contribute to the elimination of nuclei in *csn-5* mutants. Checkpoint activation is associated with an increase in aggregated SYP-1. However, while the percent of nuclei with aggregated SYP-1 is reduced by removing both checkpoints, the removal of the synapsis checkpoint (*pch-2* mutant) affects only the *csn-5* mutants. Pairing levels in *csn-5* mutants are higher than observed in *csn-2* mutants; it is possible that *csn-5* mutants attempt more to synapse (and fail) more compared to *csn-2* mutants, which leads to a robust activation of the synapsis checkpoint. PCH-2 serves as a kinetic barrier for synapsis, slowing down synapsis [71]. Therefore, it is possible that elimination of *pch-2* in the *csn-2* background increases the percent of linear SYP-1 merely by increasing the speed of assembly. Although this is possible, the removal of *cep-1*, which is not known to act like *pch-2*, has the same effect on the reduction of nuclei with SYP-1 aggregates. We propose that the reduction in fraction of nuclei with SYP-1 aggregation in a checkpoint mutant is due to the removal of the apoptotic program. This may be done directly by inducing changes in SC morphology or by activating downstream meiotic arrest providing more time for SC elongation. Alternatively, it could be done directly by preferentially eliminate nuclei with linear SYP-1.

### How CSN/COP9 regulates chromosome synapsis

There are three known examples of PC-like structures in *C. elegans* mutants: *cra-1; spo-11* double mutants [14], *pgl-1* mutants at 25°C and higher [20] and dynein mutants in early prophase [21]. Our analysis thus far is consistent with a different function for the CSN/COP9 signalosome; aggregates in *csn* mutants are found in the presence of DSBs (unlike *cra-1*), at normal growth temperatures (unlike *pgl-1* mutants], and throughout the germline (unlike dynein mutants). Therefore, we propose that CSN/COP9 participates in SC assembly in a novel manner. The signalosome's role is not merely due to promoting SYP degradation; we confirmed using several assays that *csn* mutants do not show increased SYP-1 levels.

Pathways of SC assembly involve post-translational modifications of SC proteins. These modifications could facilitate CR protein association with chromosomes and prevent their aggregation. In yeast, it was shown sumoylation promotes lateral element [72] and CR [73] assembly. Mouse SC assembly is regulated by phosphorylation of lateral element proteins [74]. In *C. elegans*, SYP-1 and SYP-2 appear to be post-translationally modified, however, the precise identities of these modifications is unknown [13]. All four SYPs contain potential sites for phosphorylation, ubiquitination, and sumoylation. In *C. elegans*, an evolutionarily conserved ubiquitin/sumo modifier [75] is linked to SC disassembly, but is not required for SC assembly [76].

The discovery that the CSN/COP9 complex is required to prevent SYP aggregation raises the question of whether CSN/COP9 is involved in post-translational modification of the SYPs to prevent their aggregation. SYP aggregates contain all four SYP proteins, hence one aggregation-prone SYP may lead to the capture of all SYPs. The CSN/COP9 complex's activity in deneddylation has been well documented, but it also possesses Ser/Thr kinase and deubiquitination associated activities [28]. CULLIN RING E3 ligases (CRLs) are the most well studied of the CSN/COP9 substrates [37], [38]. The current literature supports a model where the CSN/COP9 signalosome destabilizes the CRL complexes by deneddylation which inhibits CRL activity [39], [41]. However null signalosome mutants do not show stabilization of CRL. This suggests that both hyper and hypo-neddylation is detrimental for CRL function. This model is consistent with our findings that RNAi for neddylation and ubiquitination can partially suppress two *csn* mutant phenotypes. Moreover, we have identified an E1 NED-8 ligase (*rfl-1*) that exhibits similar SYP-1 localization defects to these of *csn* mutants. Our data also suggest that CSN/COP9 acts through CUL-4 to regulate SC assembly. If so, one role of the CSN/COP9 signalosome could be removing NED-8 to regulate CUL-4, which in turn regulates the SYPs by ubiquitination, supporting their proper assembly. In the absence of such modification, SYPs would aggregate. In this view, CSN/COP9 would repress SYPs aggregation indirectly, by inhibiting CRL monoubiquitination of SYPs which promotes SC assembly.

### CSN subunits acting outside the CSN/COP9 complex

The CSN/COP9 complex is composed of 7 to 8 subunits, depending on the organism [28]. The CSN5 subunit is responsible for the deneddylation activity of the CSN/COP9 complex, but cannot function in deneddylation outside the holoenzyme [77], [78]. Smaller sub-complexes, with variable subunit composition have been isolated as well: CSN4-7 *Arabidopsis* and *Drosophila* [79], [80] and CSN-4-5-6-8 in mammals [81]. Mutant analyses have indicated the loss of any one subunit leads to signalosome disassembly [82], [83]. The *csn* mutant phenotypes are not always identical, suggesting functions outside the CSN/COP9 signalosome for individual subunits. For example: *Drosophila csn4*, *csn5* and *csn8* mutations cause larval lethality, but they die in different larval stages [42], [68], [77], [80]. Furthermore, *S. pombe csn1* and *csn2* mutants show defects in meiotic entry and meiotic recombination, while mutants in the other subunits have no clear meiotic phenotypes [84]. CSN5 [39], [85] and CSN2 [86] are the only subunits shown to act outside CSN/COP9 *in vivo*.

We have shown that *csn-2*, *csn-5* and *csn-6* mutants all lead to defects in SC assembly, a reduction in pairing, and increase in DSB repair defects in meiotic recombination. However, the magnitude of these effects varies (see summary in Figure S7). Pairing analysis show that the *csn-2* mutant almost mimics a *syp* null mutant, while the *csn-5* and *csn-6* mutants show milder pairing stabilization defects (5S FISH) and double the numbers of designated crossovers (COSA-1) compared to the *csn-2* mutant. The SC is driving the stabilization of early prophase pairing interactions (zone 2–3), while later events (zone 6) are also promoted by the stabilizing role of crossovers, which are likely higher in the *csn-5* and *csn-6* mutant (COSA-1). Therefore, as for pairing and crossover formation, *csn-5* and *csn-6* both display milder phenotypes compared to *csn-2*. However, when examining synapsis (SYP-1) and the kinetics of repair of recombination intermediates (RAD-51), *csn-2* and *csn-6* cluster together with milder phenotypes compared to *csn-5*. It is hard to reconcile this model with a strictly linear role for the CSN/COP9 signalosome affecting synapsis, pairing, recombination progression and ending at crossover formation. It is likely that the picture is more detailed and complex. We propose that in addition to the central role of the CSN/COP9 signalosome in SC formation (which affects recombination and pairing stabilization) additional roles exist in down-regulating pairing initiation and promoting crossover formation, independently of the SC. These different roles may stem from alternative complex formation, as was shown in other organisms. One possible model would involve an inhibitory role for CSN-5-CSN-6 on pairing stabilization outside the CSN/COP9 holocomplex. CSN-5 and CSN-6 have been shown to physically interact and form a sub module in the CSN/COP9 complex [78]. We do have some support for a role for CSN-5 in down-regulating pairing initiation: as expected by this model, *csn-5* is epistatic to *csn-2* as for pairing interactions.

Identifying the precise role of each subunit and sub-complexes in meiosis will require more extensive analysis of each subunit. The work presented here is but a first step in this direction. It is intriguing that both neddylation [43] and deneddylation (this study) have such profound effects on crossovers and SC formation. These studies strengthen our claim that the balance between neddylation and deneddylation is key to accurate meiosis and these processes are likely to be evolutionarily conserved.

## Materials and Methods

### Strains

Most *C. elegans* strains were cultured under standard conditions at 20°C [87]. Several strains (in bold) were maintained at 15°C and experimentally cultured at 26°C. N2 Bristol worms were utilized as the wild-type background. The following mutations and chromosome rearrangements were used:

LGI: *cep-1(ep347), csn-2(tm2823), glh-1(gk100), hT2[bli-4(e937) qIs48]*

LGII: *pch-2(tm1458), cul-4(ok1891)*

LGIII: *rfl-1(or198)*

LGIV: *csn-5(ok1064), csn-6(ok1604), kgb-1(um3), nT1[qIs50]*

LGV: *syp-1(me17)*

The following transgenic lines were used: *meIs8(GFP::COSA-1), smIs34 [ced-1p::ced-1::GFP+rol-6(su1006)]* and *meIs9[unc-119(+)pie-1promoter::gfp::SYP-3]; unc-119(ed3)III* [88]. All strains were outcrossed 6 times except *glh-1(gk100)* which was outcrossed twice, *pch-2(tm1458)* outcrossed 5 times and *cul-4(ok1891)* which was outcrossed once.

### Analyses of the *csn* allele transcripts by RT-PCR

In order to determine if the deletions in the *csn* mutants are in-frame or out of frame, we conducted RT-PCR analyses. This was done using the Superscript III OneStep RT PCR kit (12574-026, Life Technologies) and the primers TGAATACGAAGATGATAGTGGCT and CAATACGCTCTGCCCAAACA for *csn-2* and CGAAGGTGCTTTTGCATCCTTTGG and GCAGATGGTCTTGGAACGTCTG for *csn-5*. Our analysis reveals that *csn-2(tm2823)* is an in-frame deletion. The *csn-2(tm2823)* transcript lacks exon 2, and results in a 139 amino acid deletion of the peptide sequence. Although this deletion is in-frame, 28% of the protein is missing, including half of the PAM domain. We did not assess whether the total levels of transcript in this mutant are reduced. *csn-5(ok1064)* is a deletion that includes the promoter region and half of the coding region. Translation from the first in-frame AUG will lead to 75 amino acid peptide, a deletion of 80% of the protein, including the catalytic MPN domain. We have not yet succeeded in amplifying a *csn-5* transcript form *csn-5(ok1064)*, suggesting that *csn-5(ok1064)* lacks a functional promoter. The *csn-6(ok1604)* is in frame deletion that includes deletion of half of the gene. The *csn-6(ok1604)* transcript is spliced from the middle of exon 1 to the start of exon 3, when exon 2 is skipped, resulting in deletion of coding sequence expected to lead to 193 amino acid deletion of the peptide sequence. Therefore, 45% of the protein is missing, including most of the MPN domain. We did not assess whether the total levels of transcript in this mutant are reduced.

### Immunostaining and microscopy

Adult hermaphrodites 20 h post-L4 were dissected to release gonads. DAPI and immunostaining was performed as described in [4]. For transgenic lines utilizing GFP fusions, fixation was in methanol for 1–5 minutes, then washed and prepared for microscopy as in [4]. Whole mount worms were prepared by Carnoy's Fixation. Antibodies were used at the following dilutions: α-SYP-1, 1∶500; α-SYP-4, 1∶500; α-HIM-3, 1∶500; α-HTP-3, 1∶500; α-RAD-51 1∶10,000; α-dpMPK-1 1∶500 (Sigma). The secondary antibodies used were: Alexa Fluor 488 α-mouse, Alexa Fluor 488 α-rabbit Alexa Fluor 555 α-rabbit, Alexa Fluor 568 α-goat, Alexa Fluor 568 α-guinea pig (Invitrogen), and DyLight 594 α-goat (Jackson Immunochemicals, West Grove, PA).

The images were acquired using the DeltaVision wide-field fluorescence microscope system (Applied Precision) with Olympus 100×/1.40- or 60× numerical aperture lenses. Optical sections were collected at 0.20-um increments with a coolSNAPHQ camera (Photometrics) and deconvolved with softWoRx software (Applied Precision). Gonadal and nuclei images are projections halfway through three-dimensional data stacks (Multiple 0.2-µm slices/stack), except of where full projections are indicated, and were prepared using softWoRx Explorer 1.3.0 software (Applied Precision) or FIJI [89].

Aggregates were defined as SYP signals with width larger than that of wild-type SC. When measured, even the smallest aggregates were larger than the larger SC width measured and above the average SC with plus 2 standard deviations. As indicated in the results section, these values were highly statistically significant (p≪0.001), indicating that our calling of SC aggregates was precise.

Quantitative analysis of the intensity of SYP-1 signals was performed on images taken from the same slide in the same exposure time. Images were analyzed using ImageJ [89]. This was performed under guided model option with a freehand polygon section in all Z-stacks of a particular SYP signal and to multiple gonads from each genetic background. We set a threshold of 250 for the max grey value being measured, to ensure that over-exposed images were not included in the analysis. Data for each nucleus is the sum of all the Z stakes in which the nuclei is detected on the DAPI channel. To obtain SYP-1 signal intensity, we subtracted the integrated intensity of the background of the same image from the SYP-1 integrated intensity to get the normalized integrated density [Nuclear (IntDen/Area) – Background (IntDen/Area)]. We presented the data as total integrated intensity ([Nuclear (IntDen/Area)−Background (IntDen/Area)]×Area of each nucleus) and also as IntDen/Area. The total integrated intensity is an indication of the total SYP-1 signal in each nucleus; the IntDen/Area is the average intensity of the SYP single in each nucleus. Statistical comparisons between genotypes were performed using the two-tailed Mann–Whitney test, 95% confidence interval.

### Fecundity assay

To determine the fecundity of the *csn* mutants, single L4 worms were placed on seeded NGM plates and allowed to lay eggs for a 15 hr period. These worms were then moved to a fresh NGM plate and again allowed to lay eggs. This was repeated for a three day period. Eggs were counted for each genotype examined.

### FISH and time-course analysis of chromosome pairing

The 5S FISH probe was generated as in [57] from a PCR fragment generated by amplifying *C. elegans* genomic DNA with the 5′-TACTTGGATCGGAGACGGCC-3′ and 5′-CTAACTGGACTCAACGTTGC-3′ primers. Fragments were labeled with fluorescein-12-dCTP (PerkinElmer, Waltham, MA). Homologous pairing was monitored quantitatively as in [57]. The total number of nuclei scored per zone (n) from three gonads each for wild-type, *csn-2* and *csn-5* mutants. Statistical comparisons between genotypes were performed using the Fishers Exact Test, 95% confidence interval.

### Time-course analysis for RAD-51 foci

Quantification of RAD-51 foci was performed for all seven zones composing the premeiotic tip to late pachytene regions of the germline as in Colaiacovo *et al.* (2003). The total number of nuclei (n) were scored per zone from three gonads each for wild-type, *csn-2* and *csn-5* mutants. Statistical comparisons between genotypes were performed using the two-tailed Mann–Whitney test, 95% confidence interval.

### Time-course analysis for COSA-1 foci

Quantification of GFP::COSA-1 was carried out as in [58] with zone 6 selected to be analyzed. The total number of nuclei (n) were scored in zone 6 for 5 gonads. Statistical comparisons between genotypes were performed using the two-tailed Mann–Whitney test, 95% confidence interval.

### Apoptosis

The *csn* mutants were introgressed to *ced-1*::GFP strain (*smIs34 [ced-1p::ced-1::GFP+rol-6(su1006)])* to assess apoptotic levels as per [90]. Images were taken at 60× and nuclei which displayed CED-1::GFP localization were counted as well as the total number of nuclei in the bend region (late pachytene). 10 different gonads were quantified. Statistical comparisons between genotypes were performed using the two-tailed Mann–Whitney test, 95% confidence interval.

### Western blotting

L4 homozygote larvae were picked and aged to adults. The mouse α-dpMPK-1 (Sigma) was used as a primary antibody (1∶1000). Mouse α-tubulin (1∶000; DSHB) was used as a loading control. Secondary antibodies used were α-mouse antibody conjugated to horseradish peroxidase (HRP; 1∶10,000). PBST-5% milk was used for incubation and blocking. Quantification was done on FIJI [89].

### RNAi screen

*csn-5* (B0547.1) was identified in a RNAi screen on the *akir-1* background for mutants affecting SC morphogenesis. Synchronized L1 larvae were placed on NGM+AMP+isopropyl-B-D-1-thiogalactopyranoside (IPTG) plates seeded with RNAi bacterial clones from the Ahringer *C. elegans* RNAi library [91] or pL4440 empty vector (control). Embryonic lethality was scored visually and clones exhibiting increased lethality as compared to controls were selected for replication and further analysis. We then conducted a fecundity study to determine if the reduction in live progeny was due to meiotic or developmental defects. Clones demonstrating a reduction in the number of eggs laid were selected for cytological screening.

### RNAi feeding protocols

RNAi clones are ground 6 hours–overnight in LB+ampicillin (50 ug/ml). Cultures are then seeded onto IPTG plates (see above) and left to grow overnight [91]. Either synchronized L1 or L4 larvae were placed on the plates, left to develop to adults. These adults were subjected to cytological analyses or their F1 progeny scored for viability.

## Supporting Information

Figure S1 SC lateral elements do not aggregate in *csn* mutants. A) Micrographs of HTP-3 (red) and DAPI (blue) stained wild-type, *csn-2(tm2823*) and *csn-5(ok1064)* nuclei representing the various stages of the *C. elegans* gonad. Images are projections through three-dimensional data stacks. Scale bar is 2 µm. PMT = pre-meiotic tip, TZ = transition zone, EP = early pachytene, MP = mid pachytene, LP = late pachytene. B) Micrographs of HIM-3 (red) and DAPI (blue) stained wild-type, *csn-2(tm2823*) and *csn-5(ok1064)*. Both HIM-3 and HTP-3 localization is not affected in the *csn* mutants.
(TIF)

Figure S2 SYP-4 aggregates in csn mutants. A–E′) Micrographs of SYP-4 (green) and DAPI (blue) stained wild-type, and *csn-5(ok1064)* nuclei representing the various stages of the *C. elegans* gonad. Images are projections through three-dimensional data stacks. PMT = pre-meiotic tip, TZ = transition zone, EP = early pachytene, MP = mid pachytene, LP = late pachytene. Aggregation affects all SYP-4 and likely all SYPs. F–G″) mid pachytene nuclei of wild-type, *csn-2* and *csn-5* mutants, all with transgenic GFP::SYP-3 (green or gray scale) and DAPI (blue). Scale bar is 2 µm.
(TIF)

Figure S3 P-granule component *kgb-1* does not have SYP-1 aggregation phenotype. A–F) Micrographs of SYP-1(red, grey scale) and DAPI(blue) stained wild-type (A–C),*kgb-1(um3)* (A′–F′), and *glh-1(gk100)* (A″–F″) mutant nuclei representing the various stages of the *C. elegans* gonad. Images are projections through three-dimensional data stacks. Scale bar is 2 µm. EP = early pachytene, MP = mid pachytene, LP = late pachytene. *kgb-1(um3)* and *glh-1(gk100)* are temperature sensitive alleles. Worms cultured at 26C do not exhibit SYP-1 aggregation. P-granules do not appear to be involved in the aggregation phenotype.
(TIF)

Figure S4 SYP-1 localization in *csn-2; csn-5* double mutants. Quantification of SYP-1 aggregates data from the entire gonad. Percent of nuclei with: no SYP-1 (black), linear SYP-1 (blue), aggregated SYP-1 (purple pink and red) and other (yellow), zones as in Figure 2A. n = 629.
(TIF)

Figure S5 SYP-1 is not overexpressed in *csn* mutants. A–D) Quantification of the amount of SYP-1 in the nuclei measured from IF images (see Materials and Methods). Reduced nuclear SYP-1 localization in *csn-5* mutants, while no effect is observed in *csn-2*. E) Western analysis confirming the reduction of expression of SYP-1 in *csn* mutants. Normalization values (α-SYP-1/α-HTP-3) shown values are of the experiment presented.
(TIF)

Figure S6 SYP-1 localization in response to *uba-1(RNAi)* and *ned-8(RNAi)*. SYP-1 localization throughout the germline of the indicated genotypes: A–C) *csn-2* mutants D–F) *csn-5* mutants, G–I) wild-type. Percent of nuclei with: no SYP-1 (black), linear SYP-1 (blue), aggregated SYP-1 (purple pink and red) and other (yellow), zones as in Figure 2A. Note that this RNAi was performed not to full penetrance to allow analysis of the germline (allow recovery of adults). n nuclei scored for whole gonad wild-type: with pL4440 = 1003, with *ned-8(RNAi)* = 653, with *uba-1(RNAi)* = 812. *csn-2*: with pL4440 = 2023 with, *ned-8(RNAi)* = 430, with *uba-1(RNAi)* = 1121, *csn-5*: with pL4440 = 2096, with *ned-8(RNAi)* = 441, with *uba-1(RNAi)* = 1014.
(TIF)

Figure S7 Schematics of the phenotypes observed in *csn* mutants. A) Comparison between each *csn* allele and wild-type for the indicated phenotypes on the left, B) Comparison between each *csn-2 and csn-5* allele, *csn-2; csn-5* double mutants and wild-type for the indicated phenotypes on the left.
(TIFF)

Table S1 Number of nuclei counted for FISH analyses. The numbers of nuclei counted for each zone and each genotype and the total number of nuclei for the FISH experiments.
(DOCX)

Table S2 *p*-values calculated by Fisher's Exact Test for all pairwise comparisons of FISH data. The *p*-values for pairwise genotype in each zone and for the FISH experiments.
(DOCX)

Table S3 *p*-values and total number of nuclei counted per zone for RAD-51 analyses. Top- the numbers of nuclei counted for each zone and each genotype and the total number of nuclei for the RAD-51 foci analysis. Bottom- the *p*-values for the pairwise genotype comparisons in each zone and for the RAD-51 foci analysis.
(DOCX)

Table S4 Average gonad lengths from PMT to dipotene and *p*-values for pairwise comparisons between the single mutant control and the double mutants. 1 day adult (24 hours post L4) is the standard age for examining meiotic events. 3 days adults (72 hours post-L4) were also analyzed for the genotypes indicated.
(DOCX)

Table S5 *p*-values and total number of nuclei counted in late pachytene zone for apoptotic analyses for the genotypes indicated.
(DOCX)

Table S6 *p*-values and total number of nuclei counted for RNAi experiments for *uba-1* and *ned-8* (see Figure 7) for the genotypes indicated. Analysis for SYP-1 aggregation is done using Fisher's Exact Test, while analysis for COSA-1 foci numbers is done using Mann Whitney Test. SYP-1 all gonad is all values for zones 1 through 6. % aggregates or average number of COSA-1 foci are for each pair of genotypes compared in the statistical test, by the order they appear on the top (e.g., bottom right corner: 2.8 is average number of foci for *pL4440* on *csn-5* and 5.0 is for *uba-1(RNAi)* on *csn-5*).
(DOCX)
