# Supplementary figures and images for: The CSN/COP9 Signalosome Regulates Synaptonemal Complex Assembly during Meiotic Prophase I of Caenorhabditis elegans
Source: PLoS Genet. 2014 Nov 6;10(11):e1004757. doi: 10.1371/journal.pgen.1004757 (PMC4222726; doi:10.1371/journal.pgen.1004757)

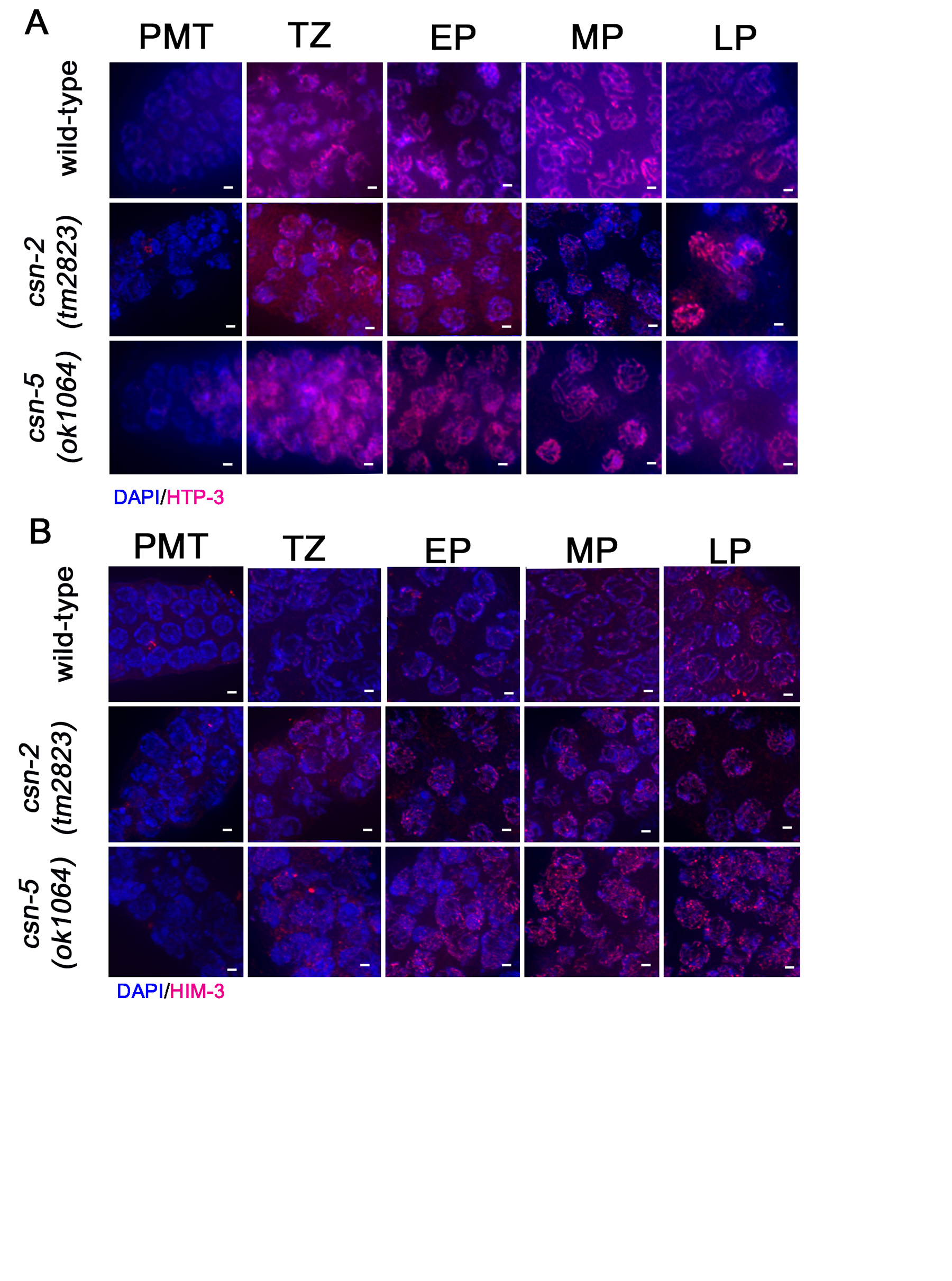

Supplement: Figure S1 — SC lateral elements do not aggregate in csn mutants. A) Micrographs of HTP-3 (red) and DAPI (blue) stained wild-type, csn-2(tm2823) and csn-5(ok1064) nuclei representing the various stages of the C. elegans gonad. Images are projections through three-dimensional data stacks. Scale bar is 2 µm. PMT = pre-meiotic tip, TZ = transition zone, EP = early pachytene, MP = mid pachytene, LP = late pachytene. B) Micrographs of HIM-3 (red) and DAPI (blue) stained wild-type, csn-2(tm2823) and csn-5(ok1064). Both HIM-3 and HTP-3 localization is not affected in the csn mutants. (TIF) [file pgen.1004757.s001.tif]

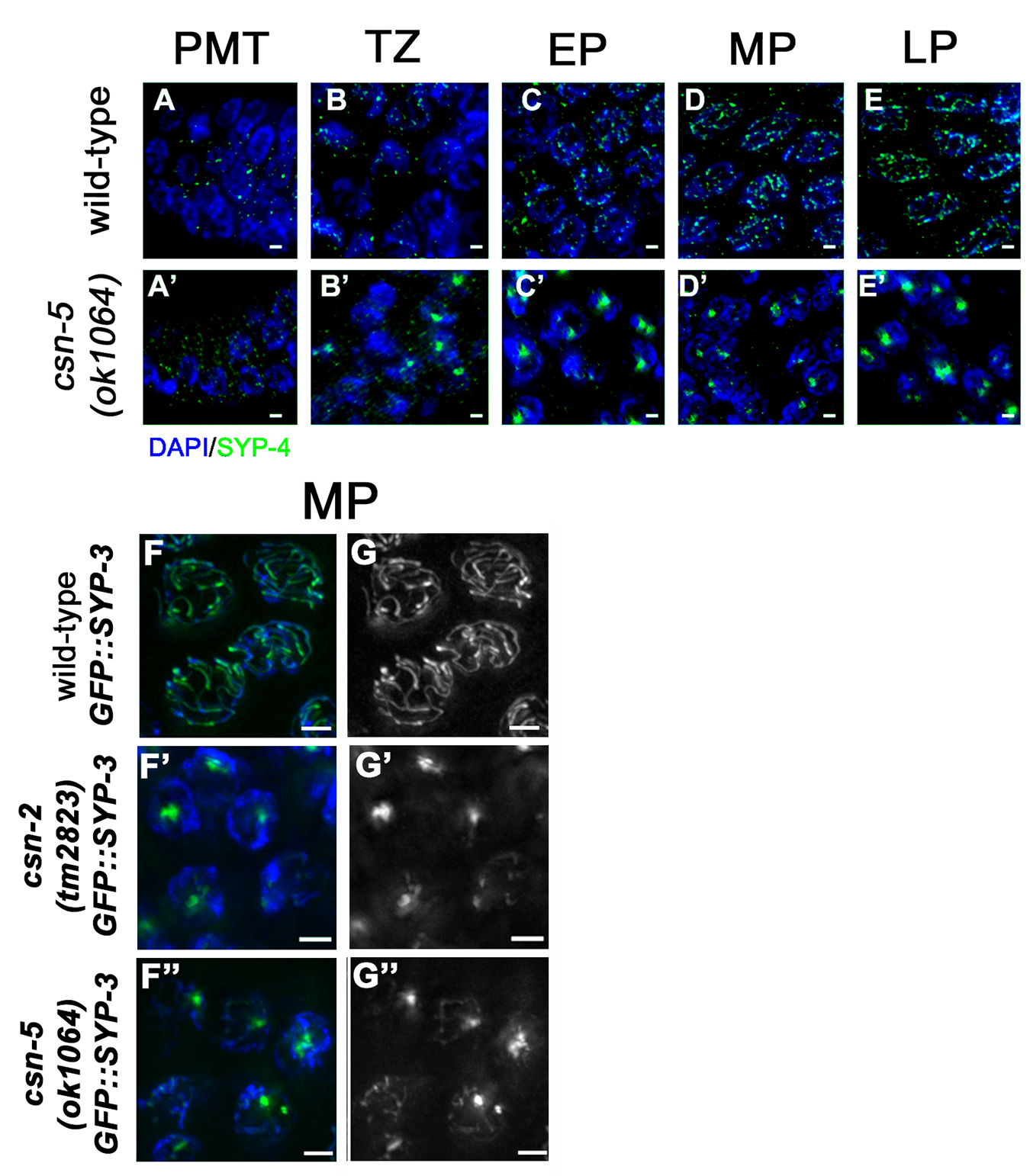

Supplement: Figure S2 — SYP-4 aggregates in csn mutants. A–E′) Micrographs of SYP-4 (green) and DAPI (blue) stained wild-type, and csn-5(ok1064) nuclei representing the various stages of the C. elegans gonad. Images are projections through three-dimensional data stacks. PMT = pre-meiotic tip, TZ = transition zone, EP = early pachytene, MP = mid pachytene, LP = late pachytene. Aggregation affects all SYP-4 and likely all SYPs. F–G″) mid pachytene nuclei of wild-type, csn-2 and csn-5 mutants, all with transgenic GFP::SYP-3 (green or gray scale) and DAPI (blue). Scale bar is 2 µm. (TIF) [file pgen.1004757.s002.tif]

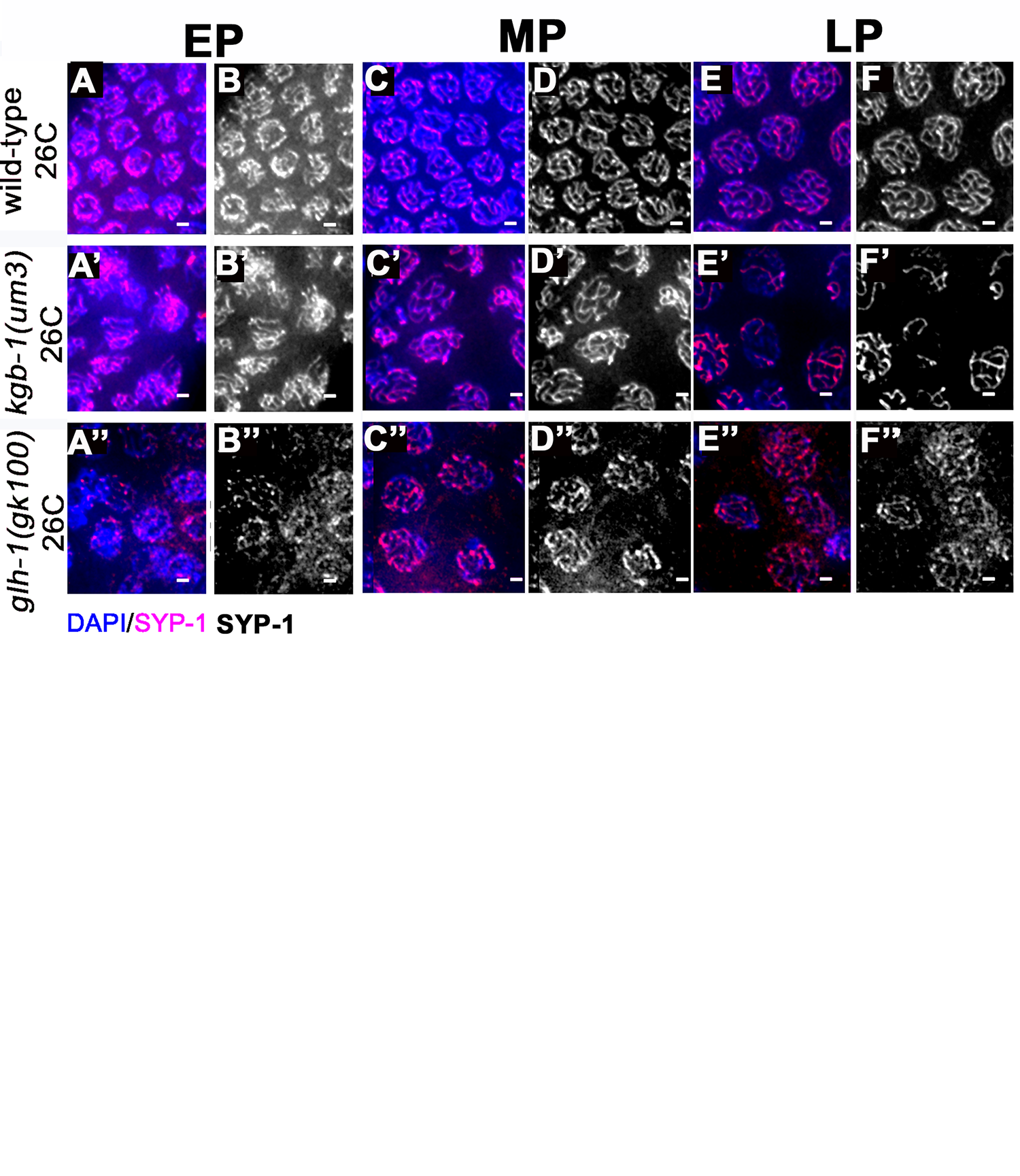

Supplement: Figure S3 — P-granule component kgb-1 does not have SYP-1 aggregation phenotype. A–F) Micrographs of SYP-1(red, grey scale) and DAPI(blue) stained wild-type (A–C),kgb-1(um3) (A′–F′), and glh-1(gk100) (A″–F″) mutant nuclei representing the various stages of the C. elegans gonad. Images are projections through three-dimensional data stacks. Scale bar is 2 µm. EP = early pachytene, MP = mid pachytene, LP = late pachytene. kgb-1(um3) and glh-1(gk100) are temperature sensitive alleles. Worms cultured at 26C do not exhibit SYP-1 aggregation. P-granules do not appear to be involved in the aggregation phenotype. (TIF) [file pgen.1004757.s003.tif]

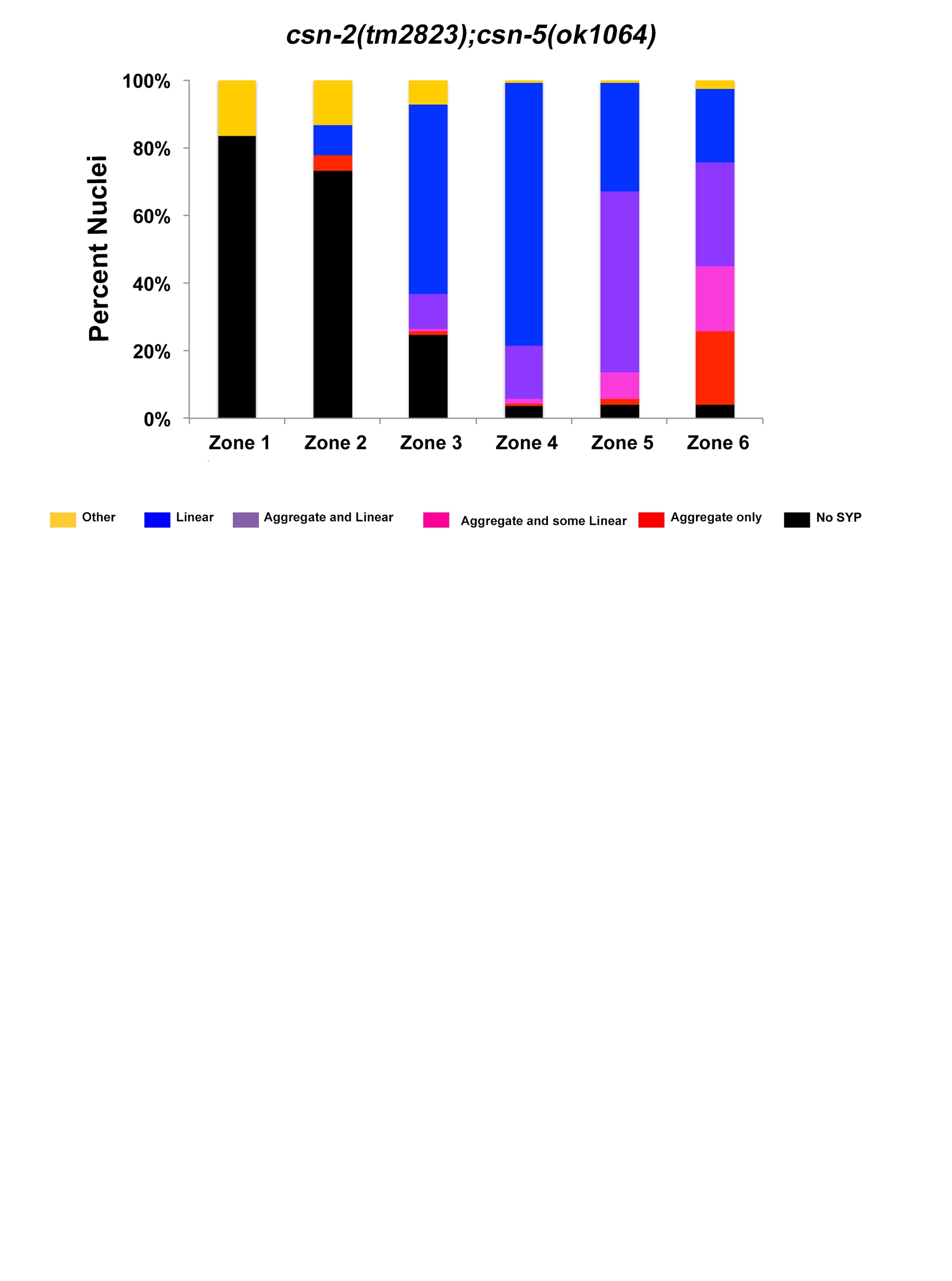

Supplement: Figure S4 — SYP-1 localization in csn-2; csn-5 double mutants. Quantification of SYP-1 aggregates data from the entire gonad. Percent of nuclei with: no SYP-1 (black), linear SYP-1 (blue), aggregated SYP-1 (purple pink and red) and other (yellow), zones as in Figure 2A. n = 629. (TIF) [file pgen.1004757.s004.tif]

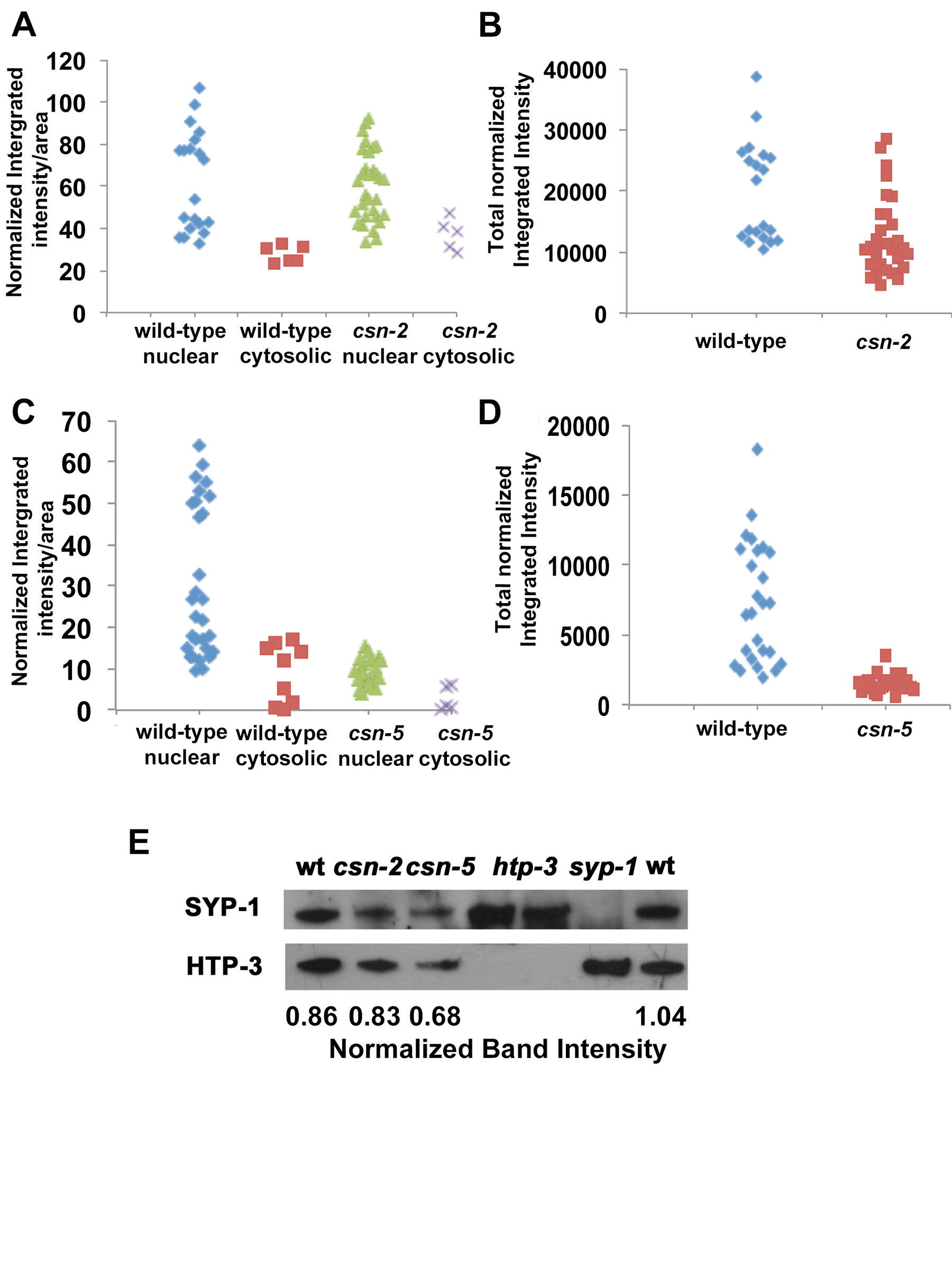

Supplement: Figure S5 — SYP-1 is not overexpressed in csn mutants. A–D) Quantification of the amount of SYP-1 in the nuclei measured from IF images (see Materials and Methods). Reduced nuclear SYP-1 localization in csn-5 mutants, while no effect is observed in csn-2. E) Western analysis confirming the reduction of expression of SYP-1 in csn mutants. Normalization values (α-SYP-1/α-HTP-3) shown values are of the experiment presented. (TIF) [file pgen.1004757.s005.tif]

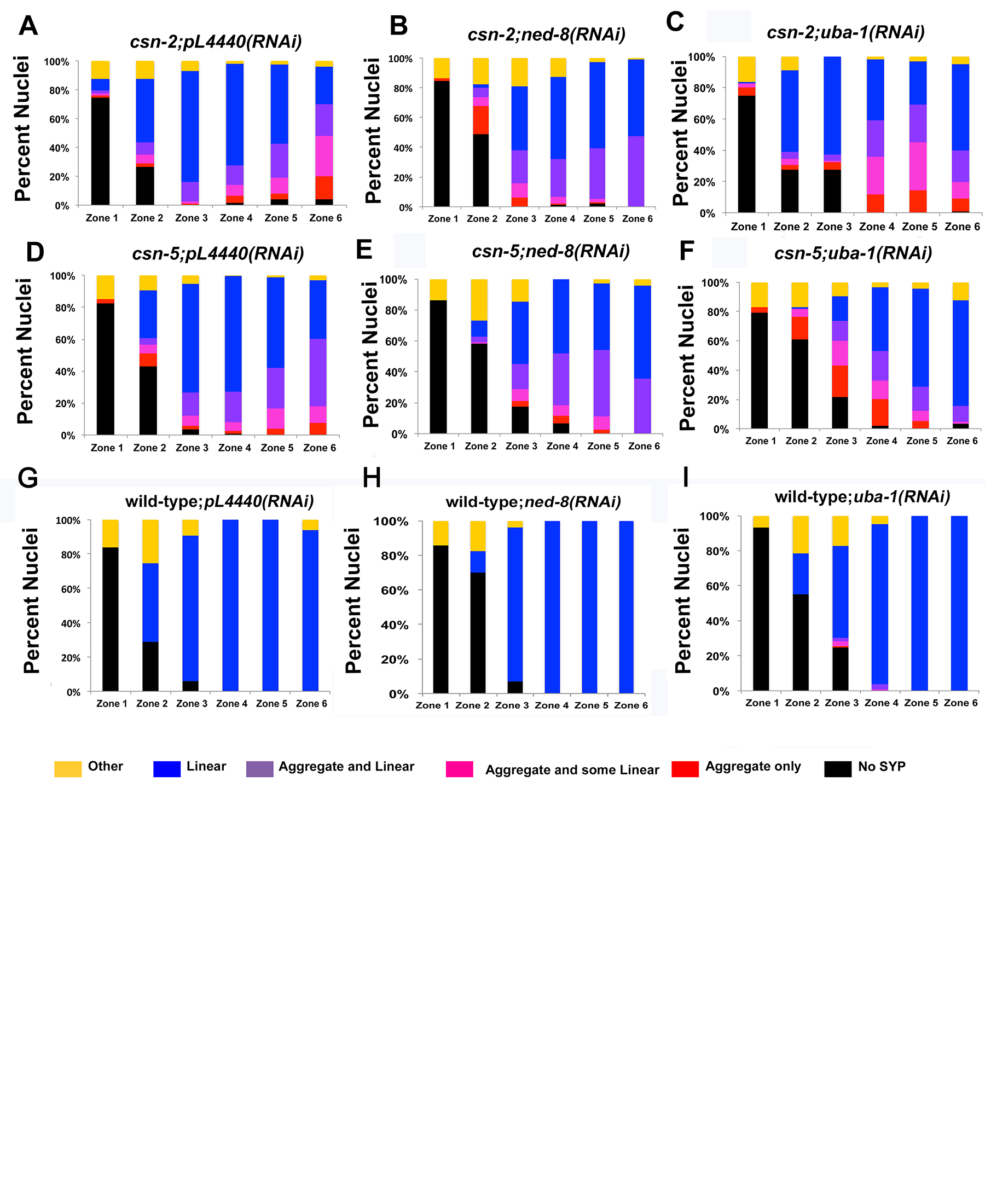

Supplement: Figure S6 — SYP-1 localization in response to uba-1(RNAi) and ned-8(RNAi). SYP-1 localization throughout the germline of the indicated genotypes: A–C) csn-2 mutants D–F) csn-5 mutants, G–I) wild-type. Percent of nuclei with: no SYP-1 (black), linear SYP-1 (blue), aggregated SYP-1 (purple pink and red) and other (yellow), zones as in Figure 2A. Note that this RNAi was performed not to full penetrance to allow analysis of the germline (allow recovery of adults). n nuclei scored for whole gonad wild-type: with pL4440 = 1003, with ned-8(RNAi) = 653, with uba-1(RNAi) = 812. csn-2: with pL4440 = 2023 with, ned-8(RNAi) = 430, with uba-1(RNAi) = 1121, csn-5: with pL4440 = 2096, with ned-8(RNAi) = 441, with uba-1(RNAi) = 1014. (TIF) [file pgen.1004757.s006.tif]

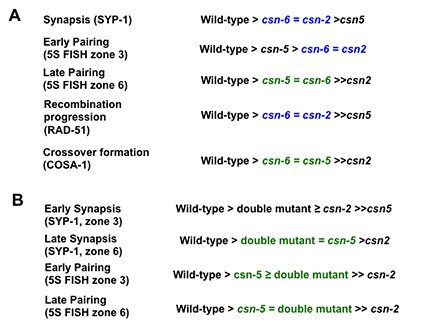

Supplement: Figure S7 — Schematics of the phenotypes observed in csn mutants. A) Comparison between each csn allele and wild-type for the indicated phenotypes on the left, B) Comparison between each csn-2 and csn-5 allele, csn-2; csn-5 double mutants and wild-type for the indicated phenotypes on the left. (TIFF) [file pgen.1004757.s007.tiff]
