# Supplementary material for: The CSN/COP9 Signalosome Regulates Synaptonemal Complex Assembly during Meiotic Prophase I of Caenorhabditis elegans
Source: PLoS Genet. 2014 Nov 6;10(11):e1004757. doi: 10.1371/journal.pgen.1004757 (PMC4222726; doi:10.1371/journal.pgen.1004757)
Supplement: Table S1 — Number of nuclei counted for FISH analyses. The numbers of nuclei counted for each zone and each genotype and the total number of nuclei for the FISH experiments. (DOCX) [file pgen.1004757.s008.docx]

**Supplemental Table 1**.

|  | **wild-type** | ***syp-1*** | ***csn-2*** | ***csn-5*** | ***csn-6*** | ***csn-2; csn-5*** |
| --- | --- | --- | --- | --- | --- | --- |
| **Zone 1** | 115 | 41 | 47 | 78 | 35 | 34 |
| **Zone 2** | 129 | 49 | 28 | 104 | 46 | 39 |
| **Zone 3** | 119 | 38 | 45 | 103 | 41 | 33 |
| **Zone 4** | 134 | 42 | 35 | 97 | 47 | 33 |
| **Zone 5** | 139 | 40 | 49 | 106 | 45 | 19 |
| **Zone 6** | 124 | 22 | 33 | 65 | 16 | 16 |
| **Total Nuclei** | 760 | 232 | 237 | 553 | 230 | 174 |
