# Supplementary material for: The CSN/COP9 Signalosome Regulates Synaptonemal Complex Assembly during Meiotic Prophase I of Caenorhabditis elegans
Source: PLoS Genet. 2014 Nov 6;10(11):e1004757. doi: 10.1371/journal.pgen.1004757 (PMC4222726; doi:10.1371/journal.pgen.1004757)
Supplement: Table S2 — p-values calculated by Fisher's Exact Test for all pairwise comparisons of FISH data. The p-values for pairwise genotype in each zone and for the FISH experiments. (DOCX) [file pgen.1004757.s009.docx]

**Supplemental Table 2**.

|  | **wt vs**  ***csn-2*** | **wt vs**  ***csn-5*** | **wt vs**  ***csn-6*** | **wt vs**  ***csn-2;csn-5*** | **wt vs**  ***syp-1*** |
| --- | --- | --- | --- | --- | --- |
| **Zone 1** | 0.146 | 0.0019 | 0.23 | 0.22 | 0.11 |
| **Zone 2** | 0.29 | <<0.0001 | 0.054 | 0.00357 | 0.218 |
| **Zone 3** | 0.0041 | 0.02 | 0.053 | 0.83 | 0.00082 |
| **Zone 4** | <<0.0001 | <<0.0001 | 0.00029 | 0.017 | <<0.0001 |
| **Zone 5** | <<0.0001 | <<0.0001 | 0.00039 | 0.03 | <<0.0001 |
| **Zone 6** | <<0.0001 | 0.0016 | 0.0038 | 0.065 | <<0.0001 |
|  |  |  |  |  |  |
|  | ***csn-2 vs csn-5*** | ***csn-2 vs csn-6*** | ***csn-2 vs***  ***csn-2;csn-5*** | ***csn-5 vs***  ***csn-6*** | ***csn-5 vs***  ***csn-2;csn-5*** |
| **Zone 1** | 0.247 | 1 | 1 | 0.33 | 0.33 |
| **Zone 2** | 0.0075 | 0.7 | 0.28 | 1 | 0.098 |
| **Zone 3** | <0.0001 | 0.56 | 0.0513 | 0.0003 | 0.07 |
| **Zone 4** | 0.35 | 0.2 | 0.058 | 0.55 | 0.19 |
| **Zone 5** | 0.24 | 0.04 | 0.061 | 1 | 0.79 |
| **Zone 6** | 0.0002 | 0.93 | 0.044 | 0.399 | 1 |
|  |  |  |  |  |  |
|  | ***csn-2 vs syp-1*** | ***csn-5 vs syp-1*** | ***csn-6 vs***  ***syp-1*** | ***csn-2;csn-5 vs syp-1*** |  |
| **Zone 1** | 1 | 0.37 | 1 | 1 |  |
| **Zone 2** | 1 | 0.001 | 0.73 | 0.2 |  |
| **Zone 3** | 0.49 | <<0.0001 | 0.19 | 0.01 |  |
| **Zone 4** | 0.77 | 0.67 | 0.34 | 0.1 |  |
| **Zone 5** | 0.21 | 0.0003 | 0.78 | 0.002 |  |
| **Zone 6** | 0.67 | 0.0099 | 0.24 | 0.03 |  |
