# Supplementary material for: The CSN/COP9 Signalosome Regulates Synaptonemal Complex Assembly during Meiotic Prophase I of Caenorhabditis elegans
Source: PLoS Genet. 2014 Nov 6;10(11):e1004757. doi: 10.1371/journal.pgen.1004757 (PMC4222726; doi:10.1371/journal.pgen.1004757)
Supplement: Table S3 — p-values and total number of nuclei counted per zone for RAD-51 analyses. Top- the numbers of nuclei counted for each zone and each genotype and the total number of nuclei for the RAD-51 foci analysis. Bottom- the p-values for the pairwise genotype comparisons in each zone and for the RAD-51 foci analysis. (DOCX) [file pgen.1004757.s010.docx]

**Supplemental Table 3**:

|  | **wild-type** | ***csn-2*** | ***csn-5*** | ***csn-6*** |  |  |
| --- | --- | --- | --- | --- | --- | --- |
| **Zone 1** | 159 | 60 | 46 | 52 |  |  |
| **Zone 2** | 209 | 70 | 49 | 87 |  |  |
| **Zone 3** | 199 | 61 | 62 | 91 |  |  |
| **Zone 4** | 177 | 56 | 47 | 75 |  |  |
| **Zone 5** | 174 | 68 | 55 | 60 |  |  |
| **Zone 6** | 142 | 47 | 49 | 36 |  |  |
| **Total Nuclei** | 1060 | 362 | 308 | 401 |  |  |
|  |  |  |  |  |  |  |
|  |  |  |  |  |  |  |
|  | **wt vs *csn-2*** | **wt vs *csn-5*** | **wt vs *csn-6*** | ***csn-2 vs csn-5*** | ***csn-2 vs csn-6*** | ***csn-5 vs csn-6*** |
| **Zone 1** | 0.1772 | <<0.0001 | 0.000173 | 0.0002 | 0.045 | 0.061 |
| **Zone 2** | 0.073 | <<0.0001 | <<0.0001 | 0.0094 | 0.014 | 0.66 |
| **Zone 3** | <<0.0001 | <<0.0001 | <<0.0001 | 0.0022 | 0.0056 | 0.98 |
| **Zone 4** | <<0.0001 | <<0.0001 | <<0.0001 | 0.0322 | 0.0001 | 0.033 |
| **Zone 5** | <<0.0001 | <<0.0001 | <<0.0001 | 0.8092 | 0.039 | 0.1 |
| **Zone 6** | <<0.0001 | <<0.0001 | 0.00044 | <<0.0001 | 0.64 | <<0.0001 |
