# Supplementary material for: The CSN/COP9 Signalosome Regulates Synaptonemal Complex Assembly during Meiotic Prophase I of Caenorhabditis elegans
Source: PLoS Genet. 2014 Nov 6;10(11):e1004757. doi: 10.1371/journal.pgen.1004757 (PMC4222726; doi:10.1371/journal.pgen.1004757)
Supplement: Table S4 — Average gonad lengths from PMT to dipotene and p-values for pairwise comparisons between the single mutant control and the double mutants. 1 day adult (24 hours post L4) is the standard age for examining meiotic events. 3 days adults (72 hours post-L4) were also analyzed for the genotypes indicated. (DOCX) [file pgen.1004757.s011.docx]

**Supplemental Table 4**

|  | **Days as adult** | **Gonad Length, PMT to diplotene** | | | **n** | ***p*-value MW** | |
| --- | --- | --- | --- | --- | --- | --- | --- |
|  |  | **Average (μM)** | **Standard error** | % of *csn* mutant |  | **wt vs *csn* mutant** | ***csn-2* vs *csn-5*** |
| **wild-type** | 1 | 307.0 | 10.23 |  | 10 |  |  |
| ***csn-2(tm2823)*** | 1 | 193.9 | 2.99 |  | 10 | 0.000157 |  |
| ***csn-5(ok1064)*** | 1 | 176.5 | 4.17 |  | 10 | 0.000157 | 0.00815 |
| ***pch-2(tm1458)*** | 1 | 311.3 | 3.53 |  | 10 |  |  |
| ***pch-2(tm1458);csn-2(tm2823)*** | 1 | 187.5 | 11.56 | 97 | 10 | 0.000157 |  |
| ***pch-2(tm1458);csn-5(ok1064)*** | 1 | 185.9 | 12.56 | 105 | 10 | 0.000157 | 0.88 |
| ***cep-1(RNAi)*** | 1 | 372.1 | 8.58 |  | 10 |  |  |
| ***csn-2(tm2823);cep-1(RNAi)*** | 1 | 190.3 | 7.38 | 98 | 10 | 0.000157 |  |
| ***csn-5(ok1064);cep-1(RNAi)*** | 1 | 185.4 | 8.7 | 105 | 10 | 0.000157 | 0.545 |
| **wild-type** | 3 | 327.5 | 4.5 |  | 10 |  |  |
| ***csn-5(ok1064)*** | 3 | 124.6 | 4.5 |  | 10 |  |  |
| ***pch-2(tm1458);csn-5(ok1064)*** | 3 | 214.3 | 19.77 | 172 | 10 | 0.00015 |  |
| ***csn-5(ok1064);cep-1*** | 3 | 212.7 | 9.88 | 171 | 10 | 0.00015 |  |
