# Supplementary material for: The CSN/COP9 Signalosome Regulates Synaptonemal Complex Assembly during Meiotic Prophase I of Caenorhabditis elegans
Source: PLoS Genet. 2014 Nov 6;10(11):e1004757. doi: 10.1371/journal.pgen.1004757 (PMC4222726; doi:10.1371/journal.pgen.1004757)
Supplement: Table S5 — p-values and total number of nuclei counted in late pachytene zone for apoptotic analyses for the genotypes indicated. (DOCX) [file pgen.1004757.s012.docx]

**Supplemental Table 5**

|  | **Number of Nuclei counted** | ***p*-value FET** | |
| --- | --- | --- | --- |
|  |  | **single vs *csn* mutant** | ***csn-2* vs *csn-5*** |
| **wild-type** | 164 |  |  |
| ***csn-2(tm2823)*** | 173 | 9.15E-48 |  |
| ***csn-5(ok1064)*** | 164 | 1.70E-59 | 0.0191 |
| ***pch-2(tm1458)*** | 171 |  |  |
| ***pch-2(tm1458);csn-2(tm2823)*** | 198 | 1.83E-47 |  |
| ***pch-2(tm1458);csn-5(ok1064)*** | 328 | 2.91E-43 | 0.0230 |
| ***cep-1(RNAi)*** | 502 |  |  |
| ***csn-2(tm2823);cep-1(RNAi)*** | 336 | 7.09E-47 |  |
| ***csn-5(ok1064);ced-1(RNAi)*** | 180 | 1.02E-42 | 0.239 |
