# Supplementary material for: The CSN/COP9 Signalosome Regulates Synaptonemal Complex Assembly during Meiotic Prophase I of Caenorhabditis elegans
Source: PLoS Genet. 2014 Nov 6;10(11):e1004757. doi: 10.1371/journal.pgen.1004757 (PMC4222726; doi:10.1371/journal.pgen.1004757)
Supplement: Table S6 — p-values and total number of nuclei counted for RNAi experiments for uba-1 and ned-8 (see Figure 7) for the genotypes indicated. Analysis for SYP-1 aggregation is done using Fisher's Exact Test, while analysis for COSA-1 foci numbers is done using Mann Whitney Test. SYP-1 all gonad is all values for zones 1 through 6. % aggregates or average number of COSA-1 foci are for each pair of genotypes compared in the statistical test, by the order they appear on the top (e.g., bottom right corner: 2.8 is average number of foci for pL4440 on csn-5 and 5.0 is for uba-1(RNAi) on csn-5). (DOCX) [file pgen.1004757.s013.docx]

**Supplemental Table 6**:

|  |  |  |  |  |  |  |
| --- | --- | --- | --- | --- | --- | --- |
|  | **wt**  ***pL4440* vs. *ned-8(RNAi)*** | **wt**  ***pL4440* vs. *uba-1(RNAi)*** | ***csn-2***  ***pL4440* vs. *ned-8(RNAi)*** | ***csn-2***  ***pL4440* vs. *uba-1(RNAi)*** | ***csn-5***  ***pL4440* vs. *ned-8(RNAi)*** | ***csn-5***  ***pL4440* vs. *uba-1(RNAi)*** |
| **SYP-1 all gonad**  **% of nuclei with aggregates,**  **pFET** | 0, 0  1 | 0, 2  <<0.0001 | 25, 33  0.0003 | 25, 28  0.097 | 29, 30  0.6643 | 29, 29  0.762 |
| **SYP-1 at Zone 6**  **% of nuclei with aggregates,**  **pFET** | 0, 0  1 | 0, 0  1 | 66, 48  0.0002 | 66, 28  <<0.0001 | 60, 44  0.0014 | 60, 12  <<0.0001 |
| **COSA-1**  **Average no of foci**  **pMWT** | 5.8, 5.4  0.9230 | 5.8, 5.6  0.9468 | 2.2, 4.0  <<0.0001 | 2.2, 3.7  <<0.0001 | 2.8, 5.0  <<0.0001 | 2.8, 5.6  <<0.0001 |
